# Requirements for establishment and epigenetic stability of mammalian heterochromatin

**DOI:** 10.1016/j.molcel.2025.08.025

**Published:** 2025-09-18

**Authors:** Antonis Tatarakis, Harleen Saini, Juntao Yu, Wenzhi Feng, Carlos A. Pinzon-Arteaga, Danesh Moazed

**Affiliations:** 1 Howard Hughes Medical Institute, Department of Cell Biology, Blavatnik Institute, Harvard Medical School, Boston, MA, USA.

## Abstract

Heterochromatic domains of DNA account for a large fraction of mammalian genomes and play critical roles in silencing transposons and genes, but the mechanisms that establish and maintain these domains are not fully understood. Here we use a CRISPR-based genetic screen to investigate the requirements for establishment and maintenance of histone H3 lysine 9 trimethylation (H3K9me3) heterochromatin. In mouse embryonic stem cells (mESCs), we show that transiently induced H3K9me3 heterochromatin is inherited for a limited number of cell divisions, independently of sequence-dependent recruitment, but becomes stable upon differentiation, concomitant with downregulation of enzymes erasing H3K9me and DNA methylation. In addition, ordered and non-redundant activities of multiple H3K9 and DNA methyltransferases, together with histone deacetylases, chromatin remodeling complexes, and RNA processing factors are required for heterochromatin maintenance. Our findings suggest that a newly acquired H3K9me3 domain can be maintained like an imprint but requires reinforcement by DNA methylation and other pathways.

## eTOC Blurb

Tatarakis et al. study how H3K9me3 heterochromatin is formed and inherited in mammalian cells. Using a synthetic heterochromatin assembly system and genetic screens, they uncover requirements for initiation and maintenance of silencing, revealing how DNA methylation and chromatin modifiers cooperate to stabilize H3K9me3 heterochromatin.

## Graphical Abstract

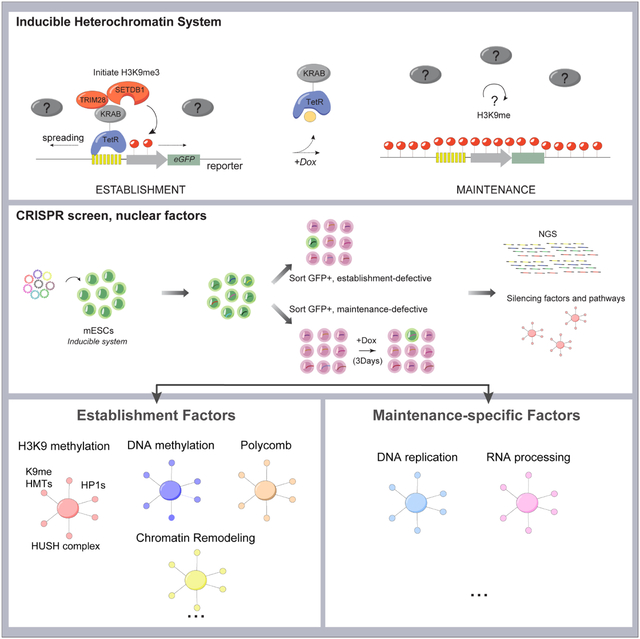

## Introduction

Heterochromatin is a conserved feature of eukaryotic chromosomes that comprises nearly half the genome in some eukaryotes and has key roles in transcriptional regulation, silencing of repetitive DNA, and genome stability^1–4^. Establishment of heterochromatic domains is initiated by the DNA sequence-dependent or RNA-mediated recruitment of histone-modifying enzymes to nucleation sites, followed by spreading of the modification to nearby regions by a read-write mechanism involving iterative nucleosome binding and modification cycles^4–6^. Studies in fission yeast have uncovered roles for specific DNA sequences in epigenetic inheritance of histone H3 lysine 9 methylation (H3K9me)-associated heterochromatin^7–10^, but whether H3K9me alone can mediate epigenetic inheritance in mammalian cells remains unknown.

In mammalian cells, H3K9me is required for the formation of constitutive heterochromatin at pericentromeric regions, telomeric repeats, rDNA repeats, interspersed repeats and also contributes to silencing of some lineage-specific genes^11,12^. Six methyltransferase enzymes are responsible for H3K9me catalysis in mammalian cells with distinct modes of action. Although the complex interplay between H3K9 methyltransferases is not well understood, deletion of all six enzymes is required to completely disrupt heterochromatin organization highlighting their unique and overlapping roles^13^. Once established, H3K9me provides binding sites for the conserved heterochromatin protein 1 (HP1) family members, which serve as a platform for recruitment of downstream effector proteins^14–16^. Additional epigenetic modifications have been linked to H3K9me heterochromatin in mammalian cells, such as DNA 5-methylcytosine (5mC), which colocalizes with H3K9me at heterochromatic domains, and is required for silencing retroviral elements in mammals^17–19^. Whether DNA methylation is generally required for silencing of H3K9me enriched regions and participates in the maintenance of H3K9me heterochromatin is unknown.

Heterochromatic domains are not transcriptionally inert and give rise to noncoding RNAs, which contribute to the assembly of heterochromatin. Studies in fission yeast, plants and animals have demonstrated the function of small RNAs and the RNA interference (RNAi) pathway in establishing heterochromatin via RNAi interactions with nascent RNA to promote the recruitment of H3K9me enzymes at specific genomic regions^20–23^. In mammalian cells, nascent repeat-associated RNAs facilitate the recruitment of H3K9me enzymes and the establishment of heterochromatin^24–28^. Such an RNA-based mechanism appears to complement the role of site-specific DNA binding factors in heterochromatin assembly. A recent study in fission yeast demonstrated that degradation of heterochromatin-associated RNAs by the rixosome complex contributes to H3K9me maintenance, further highlighting the role of RNA processing in epigenetic inheritance of heterochromatin^29^. Transcription and noncoding RNAs also contribute to the formation of mammalian heterochromatin^20–23^, but the molecular mechanism responsible for their regulation and their role(s) in heterochromatin maintenance are not fully understood.

Although previous studies have demonstrated that ectopic domains of H3K9me3 heterochromatin can be established and stably maintained in mammalian cells, the specific molecular requirements remain poorly understood^30–33^. To address this gap, we used an inducible heterochromatin assembly system to separate the establishment and epigenetic inheritance phases of heterochromatin formation. We show that in mouse embryonic stem cells (mESCs), heterochromatin can be inherited independently of underlying DNA sequence input but only for a limited number of cell divisions and that this metastable mode of inheritance becomes remarkably stable upon differentiation. Furthermore, to comprehensively assess the importance of nuclear factors in the establishment and epigenetic inheritance of heterochromatin, we performed a two-tiered CRISPR-Cas9-based genetic screen. We identified numerous factors required for heterochromatin establishment and those required solely for maintenance. Our findings identify a critical role for DNA methylation and multiple H3K9 methyltransferases, and additional chromatin and RNA processing pathways, in heterochromatin establishment and maintenance.

## Results

### Inducible system to separate establishment and maintenance of silencing in mESCs

**Figure 1.**
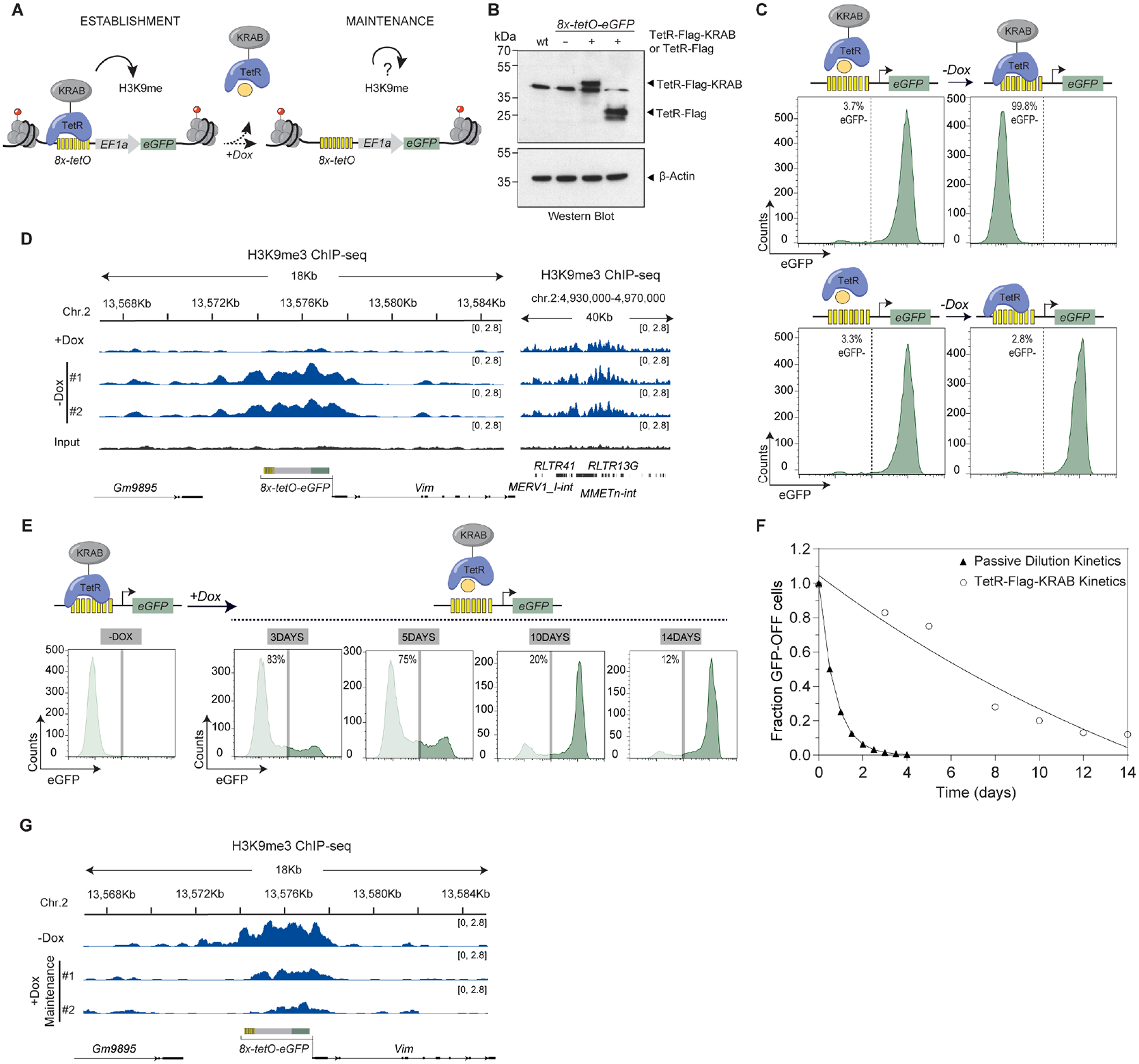
Maintenance of inducible silencing and H3K9me3 in mESCs. **(A)** Schematic of the inducible heterochromatin system. EF1a, elongation factor 1a promoter; *8x-tetO*, *tet* operator array; TetR-KRAB, TetR fused to the KRAB domain; H3K9me, tri-methyl histone H3 Lys 9; DOX, doxycycline. **(B)** Western blot of TetR-Flag-KRAB and TetR-Flag (top) in *Rosa26*-targeted mESCs lines; β-Actin (bottom) as loading control. Molecular weights are shown on the left. **(C)** Flow cytometry of eGFP expression before and 10 days after TetR-Flag-KRAB or TetR-Flag recruitment. Top, experiment schematic. % indicates eGFP-negative cells. **(D)** H3K9me3 ChIP-seq tracks at the reporter locus before (+Dox) and after (−Dox) silencing establishment. Normalized (CPM) reads in brackets. Top, chromosome coordinates; Right, ERV shown as control. **(E)** Flow cytometry of eGFP expression following Dox addition to assess silencing maintenance. **(F)** Decay of silencing during maintenance. Fraction of GFP-OFF cells at various time points after addition of Dox to cells with an established silenced state (o) and simulated data for the theoretical maintenance of the silent state assuming only passive histone dilution (▲). Exponential curve fitting was used as a guide. **(G)** Genome tracks of H3K9me3 ChIP-seq at the reporter locus (as in **D**) after establishment (−Dox) of silencing and 5 days after Dox readdition to assess maintenance. Normalized reads are presented in brackets (CPM). Top, chromosome coordinates. See also Figure S1.

To determine whether mammalian heterochromatin maintenance can be separated from the DNA sequences that initiate its establishment, we developed an inducible heterochromatin assembly system in mESCs. We fused the bacterial tetracycline repressor (TetR) to the Krüppel-associated box (KRAB) domain found in many zinc finger proteins, which recruits the H3K9 methyltransferase SETDB1 via the TRIM28/KAP1 scaffold protein to initiate silencing^34,35^. The TetR DNA binding domain targets this fusion protein to a locus that harbors its cognate DNA binding sequence, tetracycline Operator (*tetO*). Addition of doxycycline (+Dox medium) releases TetR-KRAB from *tetO* sites, allowing us to separate the sequence-dependent heterochromatin establishment phase from the sequence-independent maintenance phase (Figure 1A). To generate a reporter locus, we inserted 8 *tetO* sites (*8x-tetO*) immediately upstream of a mammalian promoter driving enhanced green fluorescence protein (eGFP) expression (Figures 1A and S1A). This reporter was inserted at a euchromatic locus, which contained active transcription-related histone modifications (H3K4me3, H3K36me3), lacked H3K9me3, and expressed mRNA in mESCs and other cell types (Figure S1A). We reasoned that such a locus would lack sequence elements that may contribute to heterochromatin maintenance or spontaneous silencing, often observed when reporter genes are inserted at gene deserts (data not shown). We further engineered mESCs to express either a Flag-tagged TetR-KRAB (TetR-Flag-KRAB) or Flag-tagged TetR as a control (TetR-Flag) (Figures 1B and S1B) and tested their effects on reporter expression by monitoring eGFP^+^ cells by fluorescence-activated cell sorting (FACS).

In the absence of Dox, eGFP was expressed in TetR or reporter-only cells but strongly silenced in TetR-KRAB cells (Figures 1C and S1C). Chromatin immunoprecipitation (ChIP) combined with high-throughput sequencing (ChIP-seq), or quantitative polymerase chain reaction (ChIP-qPCR) showed establishment of an 8- to 10-kb H3K9me3 domain spanning the reporter region in TetR-KRAB cells cultured in −Dox medium, comparable to native intracisternal A particle (IAP) and ERV loci (Figures 1D and S1D). H3K4me3 decreased to background levels in TetR-KRAB cells (Figure S1E). To assess whether the eGFP^−^ silent state was maintained without continuous establishment, Dox was added to release TetR-KRAB from *tetO* sites. The silent state persisted in ~85% of cells after 3 days in +Dox medium (~5–6 cell divisions), ~75% after 5 days (~9–10 cell divisions), ~20% after 10 days (~18–20 cell divisions), and ~15% after 14 days (~25–28 cell divisions) (Figures 1E, 1F). The decay rate of the silent state was slower than predicted for loss due to passive histone dilution, indicating an active propagation mechanism (Figure 1F). Passive dilution of histones during cell division (~12–14 hours for mESCs) would eliminate H3K9me3 within 4 days. Instead, we observed persistent silencing beyond this time frame, suggesting active maintenance during each cell cycle (Figure 1F). Consistent with this, ChIP-seq and ChIP-qPCR showed that the H3K9me3 domain was still maintained after 5 days in +Dox medium though decreased in size and magnitude (Figures 1G and S1F), matching the loss of silencing in 25% of cells. Control ChIP verified TetR-KRAB and TetR recruitment to *8x-tetO* sites in −Dox medium and release in +Dox medium (Figure S1G), with no binding at a negative control locus (*Gapdh*) (Figure S1H). To rule out residual or weak binding of TetR-KRAB to the *8x-tetO* sites, we used CRISPR-Cas9 to delete the TetR-KRAB expression cassette. Deletion prevented reporter silencing (Figures S1I, S1J). After 7 days of establishment, deleting TetR-KRAB (Figure S1K, S1L) still allowed maintenance of silencing in a subset of cells after Dox addition, similar to wild-type cells (Figure 1F and S1L), ruling out heterochromatin maintenance due to residual TetR-KRAB binding to the *8x-tetO* sites. These results demonstrate that a newly established H3K9me domain and its silent state can be maintained without sequence-dependent initiation through a limited number of mitotic cell divisions in mESCs.

### A CRISPR screen identifies factors essential for the establishment of silencing

**Figure 2.**
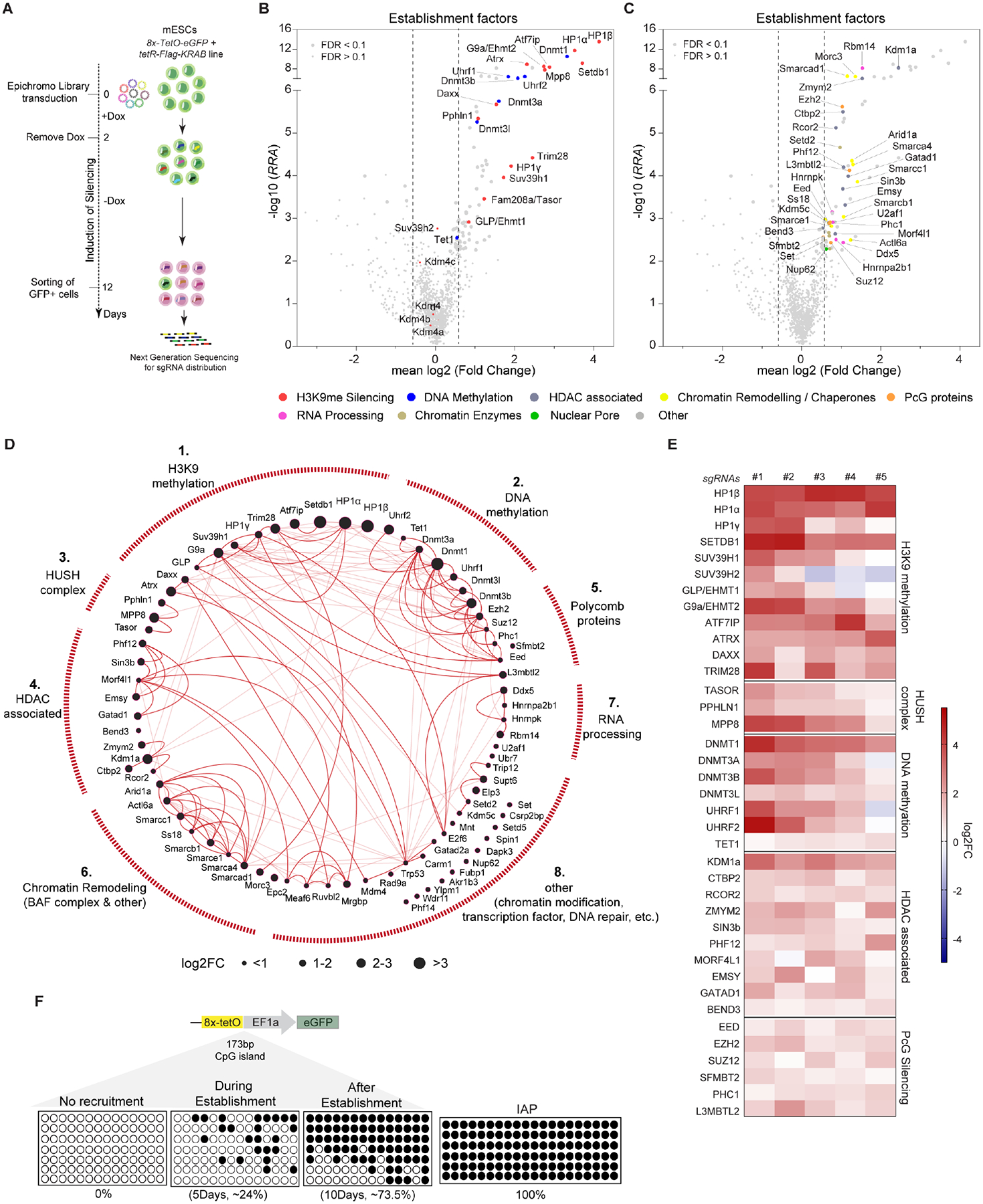
A CRISPR screen identifies genes required for the establishment of silencing. **(A)** Schematic of CRISPR screen for silencing establishment factors. **(B)** Volcano plot of the mean log_2_ fold ratios of all 5 sgRNAs targeting each gene in eGFP^+^ sorted versus unsorted cells (x axis) plotted against the negative log_10_ significant score (RRA) values (y axis) (n = two replicates). Dashed vertical black lines mark the mean log_2_ (Fold Change) −0.585 (left) and 0.585 (right). Highlighted are chromatin-associated proteins, grouped into known functional categories (color coded). Source data are provided in Table S3. **(C)** Same as in (**B**) highlighting additional functional groups. **(D)** STRING clustering of 79 factors (FDR < 0.1). Functional interactions shown with red lines. Darker red lines depict Markov clustering (MCL) interactions. **(E)** Heatmap of log_2_ fold change for all sgRNAs per gene (n = two replicates), categorized by function. **(F)** Bisulfite sequencing of DNA CpG methylation at the reporter locus before recruitment of the TetR-Flag-KRAB and during establishment. Filled and open circles represent methylated and unmethylated CpGs, respectively. Intra-cisternal A-type (IAP) used as control. % indicate methylated CpGs. See also Figures S2–S4 and Table S3.

To identify factors important for H3K9me-dependent silencing in mESCs, we performed a forward CRISPR-Cas9-based genetic screen in mESCs carrying the inducible heterochromatin system. We generated a pooled library of 5950 single guide RNAs (sgRNAs) targeting 1160 genes (5 sgRNAs/gene and 150 non-targeting controls; “EpiChromo” Library), including known chromatin regulators, DNA replication, nuclear periphery, and RNA processing factors. We also included factors identified in heterochromatin proteomic analyses but with unclear functions (Figure S2A, Tables S1, S2). Using lentiviral transduction, we introduced sgRNAs into engineered mESCs before establishment of a silent state (eGFP^+^), ensuring each cell incorporated a single sgRNA (Figure 2A). We then switched cells to −Dox medium to establish reporter gene silencing (Figure 2A). After 10 days, we isolated eGFP^+^ cells by FACS, presuming that each cell contained a mutation in a factor essential for establishment of silencing. We then performed high-throughput sequencing to assess the frequencies of the individual sgRNAs in eGFP^+^ cells, and unsorted cells for comparison. We used the Model-based Analysis of Genome-wide CRISPR-Cas9 Knockout (MAGeCK) robust ranking aggregation (RRA) algorithm for hit identification and statistical analysis (Figure 2A)^36^.

We identified 79 genes required for silencing establishment, at a 10% false discovery rate (FDR) cut-off requiring at least 3 of 5 sgRNAs per gene enriched (Figures 2B, 2C; Table S3). Gene ontology analysis revealed enrichment for heterochromatin formation, DNA methylation, histone methylation, transcriptional regulation and chromatin remodeling (Figures 2B, 2C and S2B, S2C). To assess the direct involvement of these factors in heterochromatin formation, we compared the 79 identified factors with five independent mammalian heterochromatin proteomic datasets that used distinct approaches to identify heterochromatin composition^11,37–40^. Strikingly, ~70% of our factors overlapped with these datasets^11,37–40^ (Figure S2D), a highly significant enrichment (*P*-value ≈ 7.0 × 10^−7^) relative to the expected background. This strong overlap indicates that most factors from our screen are directly associated with heterochromatin and supports the specificity of our approach. We then validated selected hits by generating lentiviral-mediated knockout mESC lines using independent sgRNAs. Consistent with the CRISPR screen results, lines targeting H3K9me methyltransferases (SETDB1, SUV39H1, SUV39H2, G9a, GLP), HP1 proteins, the HUSH complex (TASOR, MPP8, PPHLN1), the chromatin remodeler ATRX, and DNA methylation-associated enzymes and factors (DNMT1, DNMT3a, DNMT3b, UHRF1, UHRF2) showed increased expression of the eGFP reporter relative to mESCs with control sgRNAs or sgRNAs targeting the *Taf3* or *Brd7* genes, which are involved in transcription and were not identified in the screen (Figures S2E, S2F). RT-qPCR analysis confirmed TetR-KRAB expression levels remained unchanged in these lines, ruling out indirect effects via altered expression of the fusion protein (Figure S2G).

The 79 factors were grouped into 8 clusters based on functional interactions in the STRING database^41^ (Figure 2D). Cluster 1 included proteins associated with H3K9 methylation, HP1 proteins (HP1α, HP1β, HP1γ), H3K9 methyltransferases (SETDB1, G9a/EHMT2, GLP/EHMT1, SUV39H1), SETDB1 interaction partners (ATF7IP and KRAB-associated protein 1, TRIM28/KAP1), and the ATRX-DAXX histone chaperone complex (Figures 2B–2E and S2H). Notably, *Suv39h2* mutations showed weaker silencing defects (FDR = 0.18, 2 of 5 sgRNAs enriched) (Figure 2E). ChIP-qPCR analysis for SETDB1, SUV39H1, and G9a before and after establishment revealed that all three enzymes were bound to the reporter locus during establishment, supporting their direct involvement in silencing (Figure S2I). Cluster 2 included DNA methyltransferases (DNMT1, DNMT3a, DNMT3b, DNMT3L), and UHRF1 (Figure 2D). Consistent with this, 5-methylcytosine (5mC) bisulfite sequencing at the promoter of the reporter gene showed a progressive increase in DNA methylation, reaching ~75% CpG methylation by day 10 (Figure 2F). Cluster 3 included HUSH components (FAM208a/TASOR, PPHLN1, MPP8) (Figures 2D, 2E and S2H).

To dissect the roles of factors in clusters 1–3 in H3K9me3 establishment, we performed targeted knockdowns and assessed H3K9me3 levels at the reporter locus during early (first 2 days) and late (7–10 days) stages (Figures S3A, S3G). Western blot confirmed efficient knockdown of the targeted factors (Figures S3B, S3H). Strikingly, SETDB1 depletion early completely abolished H3K9me3 deposition, demonstrating its absolute requirement for initiating silencing (Figure S3C). By contrast, knockdown of SUV39H1, SUV39H2, G9a, and GLP had no early effects on H3K9me3 levels (Figure S3D–E). To test potential redundancy among these enzymes, we performed double knockdowns of SUV39H1 and SUV39H2 or G9a and GLP (Figure S3B). Even under these conditions, H3K9me3 establishment at the early stage remained unaffected, further underscoring the unique and non-redundant role of SETDB1 in initiating H3K9me3 deposition (Figure S3D–E). Similarly, knockdown of DNMT3b, DNMT3L, or TASOR had no effect on H3K9me3 levels at this stage, suggesting that these factors were not required for the initial establishment of H3K9me3 (Figure S3B, S3F). We next tested whether these factors contribute to accumulation of H3K9me3 during the late stages of establishment (7–10 days after silencing onset) (Figure S3G). SETDB1 knockdown still reduced H3K9me3, though not completely, suggesting partial compensation (Figure S3I). Indeed, knockdown of SUV39H1 and SUV39H2, either individually or in combination, reduced H3K9me3 levels across the entire reporter locus, unlike at the early stage (Figure S3J). Notably, the double knockdown of SUV39H1/SUV39H2 further reduced H3K9me3, indicating that they play partially redundant roles in accumulation of higher levels of H3K9me3 following its initial deposition by TetR-KRAB-mediated recruitment of SETDB1 (Figure S3J). Knockdown of G9a or GLP reduced H3K9me3 levels, particularly near the *tetO* site, suggesting a localized role, while double knockdown of G9a/GLP did not further reduced levels, indicating some functional overlap (Figure S3K). DNMT3L and TASOR knockdowns also reduced H3K9me3 levels, whereas DNMT3b knockdown had no significant effect (Figure S3L). Overall, these results suggest a hierarchical model for H3K9me3 establishment at the inducible reporter locus involving the non-redundant function of multiple H3K9me3 enzymes working in a temporal manner. SETDB1 is uniquely required for the initial deposition of H3K9me3 during the early stages of silencing (consistent with its recruitment via TetR-KRAB). By contrast, SUV39H1, SUV39H2, G9a, GLP, TASOR, and DNMT3L act later to promote accumulation of H3K9me3.

Cluster 4 contained proteins associated with histone deacetylation (HDACs) enzymes. We identified components of the SIN3/HDAC complex (SIN3b, MORF4L1, PHF12, EMSY, and GATAD1), and the coREST/HDAC complex (RCOR2, KDM1A/LSD1, ZMYM2, and CTBP2) (Figures 2C–2E, S2H, Table S3). Notably, HDACs themselves were not uncovered as positive hits (Figure S4A). Since heterochromatic domains are generally hypoacetylated, and the role of HDACs and their complexes in heterochromatin formation and silencing is well documented^23,42^ this likely reflects redundancy among the many HDAC enzymes in mESCs.

Cluster 5 included Polycomb-dependent silencing factors (SUZ12, EZH2, EED, PHC1, SFMBT2, L3MBTL2), though with weaker effects than the core H3K9me and DNA methylation proteins (Figures 2C–2E and S2H)^43^. A functional interplay between H3K9me and DNA methylation with Polycomb-mediated silencing has been previously reported^44–47^. To test whether the reporter locus gains H3K27me3 after silencing establishment, we performed H3K27me3 ChIP-qPCR and found increased enrichment (Figure S4B). We further performed ChIP-qPCR for SUZ12, the core component of the PRC2 complex, and observed that its binding to the reporter locus was also greatly enriched during establishment (Figure S4C). These results suggest that under our experimental conditions the H3K27me3 pathway contributes to the establishment of silencing at the reporter gene.

Cluster 6 contained BAF chromatin remodeling complex components (SMARCA4, SMARCB1, SMARCE1, ACTl6A, ARID1A, SMARCC1, SS18) and other remodelers including the Swi/Snf-related protein SMARCAD1 and the MORC family CW-type zinc finger protein MORC3 (Figures 2C–2E and S4D), some of which have been previously implicated in retroviral silencing^48–51^. Cluster 7 included proteins associated with pre-mRNA processing. For example, DDX5 (RNA helicase involved in pre-mRNA splicing, which also acts as a corepressor together with HDAC1)^52^, RBM14 (RNA binding protein regulator of splicing and transcriptional corepressor), HNRNPK and HNRNPA2B1 (pre-mRNA binding proteins), and U2AF1 (U2 snRNA auxiliary factor 1) were all required for reporter gene silencing (Figures 2C–2D and S4E). However, remodeling complexes and pre-mRNA processing factors play widespread roles in regulation of gene expression and indirect effects on silencing of the reporter locus cannot be ruled out. Cluster 8 represented proteins affecting histone methylation and chromatin dynamics, including KDM5C (transcription-associated demethylase) and SET, a subunit of the inhibitor of acetyltransferase activity (INHAT) complex proposed to inhibit histone acetylation and promote heterochromatin assembly (Figures 2C–2D and S4F)^53–55^.

### Identification of genes required for epigenetic inheritance of silencing

**Figure 3.**
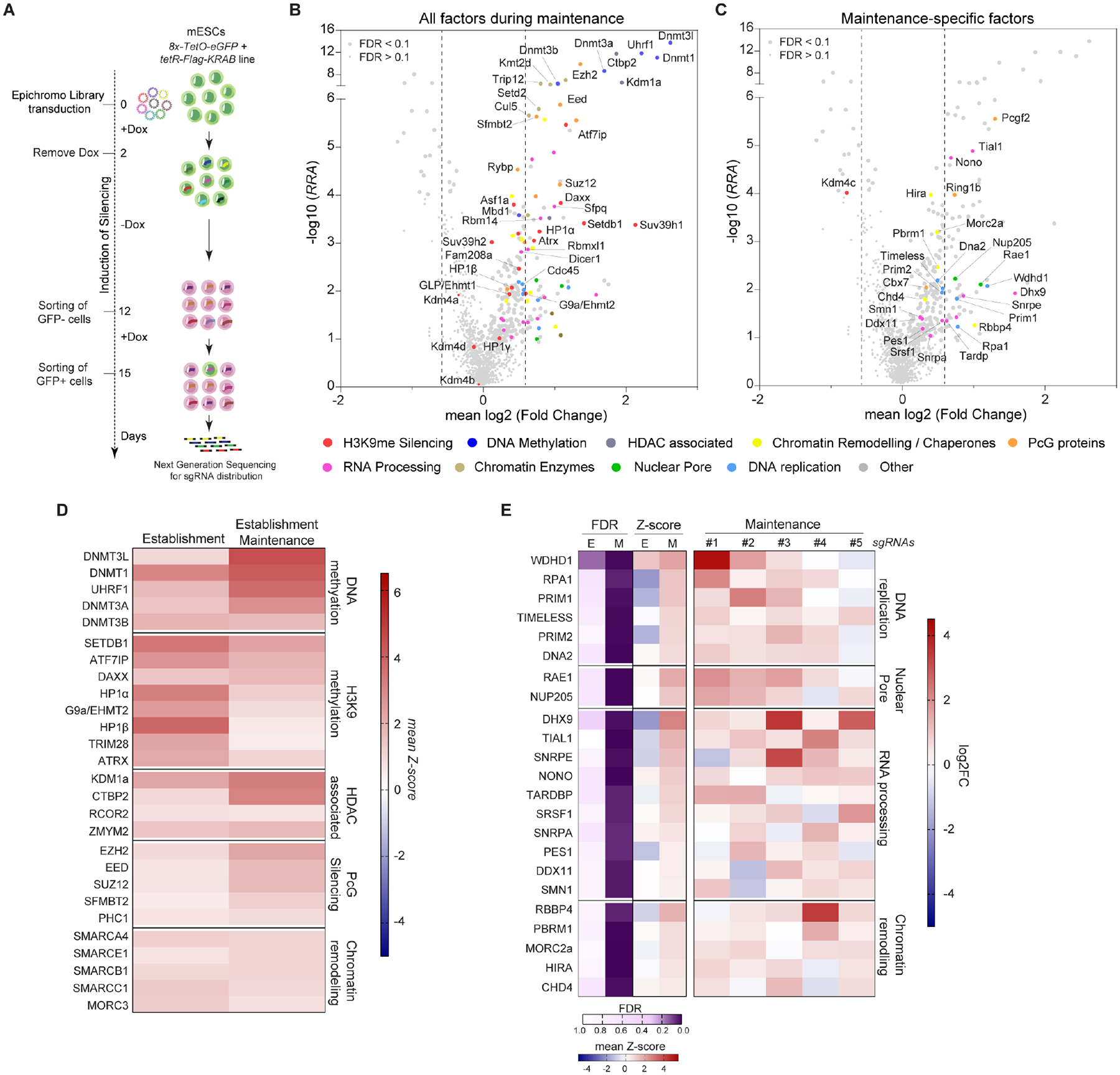
Identification of genes required for epigenetic inheritance of silencing. **(A)** Schematic of CRISPR screen to identify silencing maintenance factors. **(B)** Volcano plot of gene hits required for maintenance plotted as in Figure 2B. Selected chromatin-associated proteins are highlighted (color coded). Source data are provided in Table S4. **(C)** Same as **B** highlighting maintenance-specific proteins. **(D)** Heatmap of gene-level Z-scores (mean of 5 sgRNAs) for factors affecting both establishment and maintenance (n = two replicates). **(E)** Heatmaps of maintenance-specific genes. Left, gene-level FDR and mean Z-scores of all 5 sgRNAs per gene during establishment (E) or maintenance (M). Right, log_2_ fold change of individual sgRNAs (n = two replicates). See also Figure S5, Table S4.

We next screened for factors required for sequence-independent maintenance of silencing, which mimics epigenetic inheritance during DNA replication and across cell division, where H3K9me3 must be maintained without continuous input from sequence-specific factors, akin to developmental silencing where initiating signals are transient but silencing endures. Ten days after silencing was established, we transduced mESCs carrying the inducible silencing system with the EpiChromo library. eGFP^−^ cells (silenced) were isolated by FACS to ensure that only cells with established heterochromatin were included in the maintenance screen (Figure 3A). These cells were then switched to +Dox medium (to release TetR-KRAB from the *8x-tetO* sites) for 3 days and eGFP^+^ cells were collected by FACS. High-throughput sequencing identified sgRNAs enriched at the maintenance stage. Since sgRNAs that targeted specific genes caused only partial loss of silencing during the establishment screen and genes involved in establishment may continue to play roles in maintaining silencing (Figure 2B, 2C and S2E), we expected to identify some factors present in both screens at the establishment and maintenance stage. Nonetheless, this screen allows us to uncover any maintenance-specific factors, loss of which does not affect initial silencing but impairs maintenance upon release of TetR-KRAB repressor.

This screening strategy identified 121 candidate genes required for reporter silencing during the maintenance phase (10% FDR, at least 3 of 5 sgRNAs per target enriched in eGFP^+^ cells, log2 fold change >0.585) (Figures 3B, Table S4). As expected, 38 of these genes overlapped with establishment hits, although some had stronger loss of silencing during the maintenance screen, while 83 were specifically required for maintenance (Figures 3D and S5A–S5E). To increase confidence that these were truly maintenance-specific, we reexamined the establishment screen using a more relaxed cutoff (FDR < 20%) and found that 19 of the 83 showed modest effects during establishment (Figures S5B, S5F). To define a high-confidence subset of factors that were specifically required for silencing during the maintenance phase, we excluded these 19 genes and retained 64 genes as strong maintenance-specific factors (Figures 3C and S5B).

Notable maintenance-specific factors included (1) components of the DNA replication machinery, such as WDHD1, RPA1, PRIM1, PRIM2, DNA2 and TIMELESS; (2) nuclear periphery proteins, including Rae1 and NUP205; (3) RNA processing factors, and (4) chromatin remodelers, including the nucleosome remodeling and deacetylase (NuRD) complex components, RBBP4 and CHD4, the BAF subunit PBRM1, HUSH and H3K9me3-interacting protein MORC2a, and the histone chaperone HIRA (Figures 3C, 3E and S5E). Additional candidates included condensin components, co-repressor and DNA binding proteins, and DNA repair factors (Figure S5G). To evaluate the direct involvement of the maintenance-specific factors in heterochromatin formation, we compared them to five independent mammalian heterochromatin proteomic datasets^11,37–40^. 37 out 64 maintenance-specific factors identified in our screen overlapped with those identified in these datasets, representing a statistically significant enrichment (*P*-value ≈ 0.0102) for proteins that physically interact with heterochromatin (Figure S5H). These findings suggest that H3K9me3 heterochromatin maintenance in mammalian cells not only requires the core H3K9me and DNA methylation enzymes, but also depends on replisome-associated proteins, nuclear periphery proteins, RNA processing factors, and chromatin remodeling complexes, many of which have uncharacterized or previously underappreciated roles.

### Epigenetic maintenance of silencing is stabilized in differentiated cells

**Figure 4.**
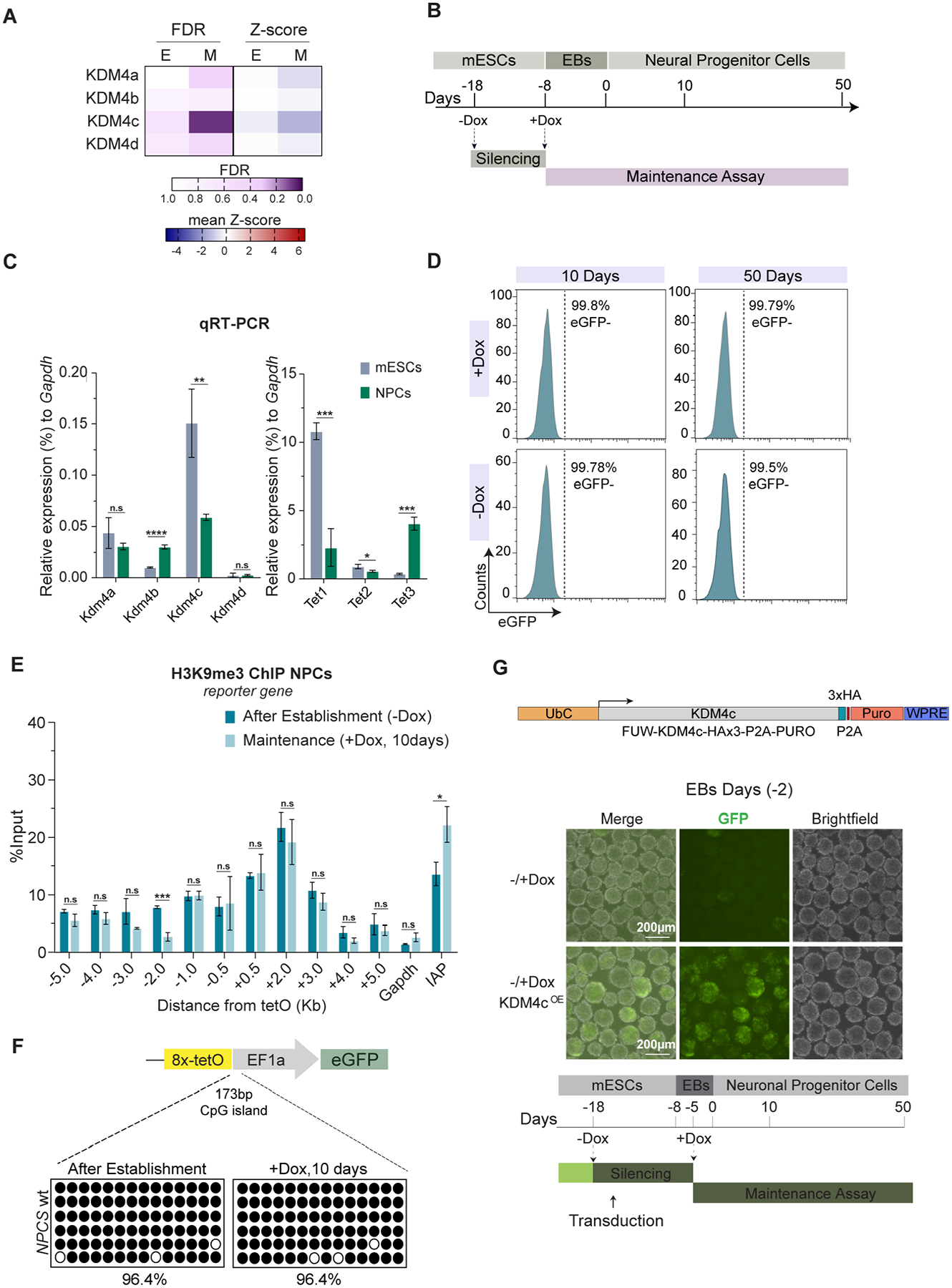
Epigenetic maintenance of silencing is stabilized upon differentiation. **(A)** Screen results for KDM4 demethylases during establishment (E) or maintenance (M), shown as in Figure 3D. **(B)** Schematic of mESCs to embryonic bodies (EB) and neural progenitor cells differentiation, and the maintenance silencing assay outline. **(C)** qRT-PCR of KDM4 demethylases and TET hydroxylases expression in mESCs and NPCs, relative to *Gapdh* (n = 3). Mean values are shown. Error bars, standard deviation (SD); *P*-values calculated with two-tailed unpaired Student’s t-tests are indicated. n.s., not significant (*P* > 0.05), *P < 0.05, **P < 0.01, ***P < 0.001. **(D)** Flow cytometry of eGFP expression in NPCs differentiated from silenced mESCs as shown in panel **C**, cultured with (+Dox) or without (−Dox). % indicate eGFP^−^ cells. **(E)** H3K9me3 ChIP-qPCR at the reporter locus in NPCs cultured without (After Establishment, −Dox) or with (Maintenance, +Dox) doxycycline. *Gapdh* and *IAP*, used as controls for euchromatin and heterochromatin H3K9me3 levels respectively. Error bars, standard deviation (SD); n = 3 replicates. *P*-values calculated with two-tailed unpaired Student’s t-tests are indicated. n.s., not significant (*P* > 0.05), *P < 0.05, **P < 0.01, ***P < 0.001. **(F)** Bisulfite sequencing of DNA CpG methylation at the reporter locus in NPCs cultured in the absence (After Establishment, −Dox) or presence (Maintenance, +Dox) of doxycycline. % indicate methylated CpGs. **(G)** Schematic of KDM4c targeting vector in mESCs. Epifluorescence images of EBs with or without KDM4c overexpression, 3 days after Dox addition during differentiation, of silenced (−Dox) mESCs. Experimental outline shown. See also Figure S6.

Although the TetR-KRAB-induced silent state could be maintained for multiple cell divisions in mESCs, it decayed over time (Figure 1F). In fission yeast, heterochromatin maintenance is opposed by the anti-silencing factor Epe1, a Jumonji (JmjC) family putative demethylase^7,8^. Our genetic screen in mESCs showed that mutation of the JmjC demethylase JMJD2c/KDM4c enhanced maintenance of the silent state without affecting establishment (Figures 2B, 3C, and 4A). In mESCs, H3K9 demethylases and 5mC hydroxylases are highly expressed, but reduced upon differentiation (Figure S6A)^56–59^. To test whether silencing maintenance was affected during differentiation, we first established the eGFP^−^ silent state in mESCs in −Dox medium and then differentiated them into neural progenitor cells (NPCs) in +Dox medium (Figures 4B). Differentiation was validated by qRT-PCR of pluripotency and NPC marker genes (Figure S6B) and showed decreased KDM4a/4c and TET1/2 expression in NPCs (Figure 4C). Cells were maintained in +Dox medium to ensure TetR-KRAB release. FACS analysis revealed that the silent state persisted in 100% of NPCs after 10 days (~10 cell divisions) and 50 days (~50 cell divisions) of growth in +DOX medium (Figure 4D). ChIP-qPCR confirmed that the H3K9me3 domain established in mESCs was maintained in NPCs in both −Dox medium (TetR-KRAB recruited at the reporter locus) (Figure S6D), and +Dox medium (TetR-KRAB released from the *tetO* sites) (Figure 4E). Bisulfite sequencing showed an increase in CG methylation in NPCs relative to mESCs with ~96% of the CG dinucleotides methylated and fully maintained following the release of TetR-KRAB (Figures 2F and 4F). Differentiation without prior establishment did not induce reporter silencing as almost all NPCs retained active reporter expression (Figure S6C). These results suggest that DNA sequence-independent epigenetic inheritance of heterochromatin is greatly stabilized in differentiated cells and correlates with increased H3K9me3 and DNA CpG methylation, coincident with reduced expression of H3K9 demethylases and 5mC hydroxylases.

To determine whether reduced KDM4c expression during differentiation contributes to the enhanced heritability of heterochromatin in NPCs, we overexpressed wild-type KDM4c (KDM4c^OE^) in mESCs and induced differentiation into NPCs (Figure S6E). Flow cytometry confirmed that ~90% of cells expressed HA-tagged KDM4c cells, with unchanged SOX2 levels, indicating no effect on pluripotency (Figure S6E). Overexpression of KDM4c resulted in loss of reporter silencing in a small fraction of cells (Figure S6E). After establishing silencing in mESCs (−Dox, 10 days), we differentiated them via embryonic body (EB) formation (Figure 4G), adding Dox during the EB stage. Three days later, KDM4c overexpressing cells showed partial loss of reporter silencing compared to controls (Figures 4G and S6F), similar to effects on endogenous heterochromatin (Figure S6G). Altogether, these results support the idea that the increased heritability of heterochromatin in differentiated cells is partly due to reduced expression of KDM4c. However, the relatively modest effect of KDM4c overexpression suggests that additional mechanisms in addition to H3K9me3, such as DNA methylation, also contribute to the stabilization of the silent state in differentiated cells.

### Deletion of Uhrf1 diminishes epigenetic maintenance of imprinted H3K9me

**Figure 5.**
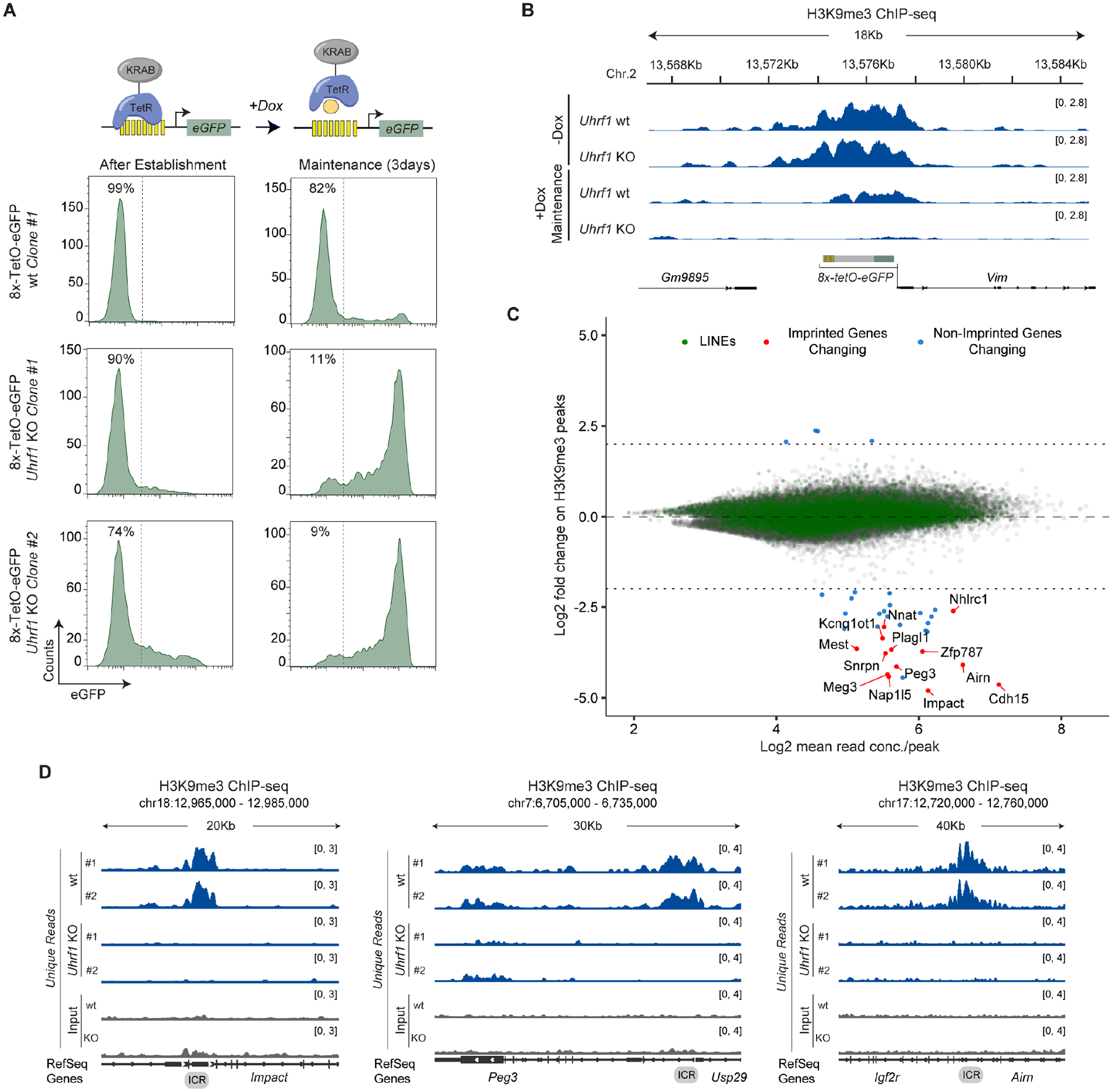
Deletion of *Uhrf1* diminishes epigenetic maintenance of H3K9me. **(A)** Flow cytometry of eGFP expression in wild-type (wt) and *Uhrf1* KO mESCs cultured without doxycycline for 12 days (After Establishment) and three days after Dox re-addition. % indicate eGFP^−^ cells. **(B)** H3K9me3 ChIP-seq at the reporter locus after establishment (−Dox) and maintenance for five days (+Dox) in wt, and *Uhrf1* KO mESCs. Normalized reads in brackets. Top, chromosome coordinates. **(C)** Scatterplot of 98,533 DiffBind consensus peaks showing mean H3K9me3 read counts in wt (x-axis) and log_2_ ratio of H3K9me3 enrichment in KO versus wt mESCs (y-axis). Dotted lines indicate log2 ratios −2 and 2. Colors: all H3K9me3 peaks (grey), peaks overlapping annotated LINE elements (green), peaks with decreased H3K9me3 in *Uhrf1* KO cells overlapping with imprinted genes (red) and peaks with decreased H3K9me3 in *Uhrf1* KO cells overlapping with annotated non-imprinted genes (blue). Source data are provided in Table S5. **(D)** H3K9me3 ChIP-seq at representative imprinted loci in *8x-tetO-eGFP* wild-type (wt) and *Uhrf1* KO mESCs. ICRs are indicated (grey boxes). See also Figure S7 and Tables S5–S6.

Our genetic analysis revealed requirements for DNA methylation associated factors in both the establishment and maintenance of H3K9me-dependent silencing. Among them, UHRF1, an E3 ubiquitin ligase that interacts with DNMT1 and is essential for DNA methylation maintenance, emerged as a top candidate^60,61^. To investigate its role in epigenetic silencing, we used CRISPR-Cas9 to knock out *Uhrf1* in mESCs carrying the inducible heterochromatin system, resulting in UHRF1 protein loss (Figure S7A). Two independent *Uhrf1* KO clones showed a moderate to weak effect on silencing establishment at the reporter (Figure 5A). However, after 3 days in +Dox medium, only 9–11% of *Uhrf1* KO cells maintained silencing, a dramatic ~8-fold reduction of silencing relative to wild-type cells (Figure 5A). ChIP-seq and ChIP-qPCR showed that H3K9me3 enrichment did not change at the reporter locus in *Uhrf1* KO cells during establishment (Figures 5B and S7B), but CpG methylation was reduced (23%) compared to wild-type cells (74%) (Figure S7D). Notably, during maintenance, H3K9me3 was lost in *Uhrf1* KO cells, suggesting a requirement for high levels of DNA CpG methylation in the epigenetic inheritance of H3K9me3 (Figures 5B and S7C). Together, these results demonstrate a critical role for UHRF1 and DNA methylation in DNA sequence-independent maintenance of H3K9me.

We then examined the role of UHRF1 in H3K9me3 maintenance at endogenous heterochromatic regions by comparing H3K9me3 ChIP-seq analysis in wild-type and *Uhrf1* KO mESCs. Genomic regions with H3K9me3 were annotated using Epic2 peak caller^62^ and DiffBind^63^ quantified differences across 98,533 consensus peaks (Table S5). We found 534 peaks with increased H3K9me3 and 185 peaks with decreased levels (FDR < 0.05) (Figure 5C; Table S5). Among the latter, a fraction of the peaks (~17%, 32/185) displayed a substantial loss of H3K9me3 enrichment (> 4-Fold) (Figure 5C; Table S5). Many overlapped imprinting control regions (ICRs), which regulate parent-of-origin-specific expression of certain genes^64^. Although, it has previously been shown that UHRF1 is responsible for the maintenance of DNA methylation and silencing at ICRs^61^, our analysis showed that *Uhrf1* KO resulted in a striking decrease in H3K9me3 at the *Impact, Peg3, Airn, Cdh15, Nap1l5, Meg3*, *Snrpn, Plagl1, Mest, Nnat, Nhlrc1, Zfp787* and *Kcnq1ot1* imprinting clusters (Figures 5C and 5D). After closer examination, additional imprinted loci that did not meet our strict statistical thresholds were found to have lost H3K9me3 in *Uhrf1* KO cells, including *Nespas, Inpp5f, Rasgrf1, Peg13* and *H19* regions. Thus, imprinted loci display a dependence on UHRF1 and DNA methylation, similar to the reporter locus.

Most H3K9me3 peaks overlap with repetitive regions. To assess UHRF1’s role in maintenance of H3K9me3 at repetitive genomic regions, we compared the total number of H3K9me3 ChIP-seq reads aligning to each annotated repeat element in *Uhrf1* KO and wild-type cells (Table S6). Apart from a decrease at the Pol I-transcribed region (LSU-rRNA, SSU-rRNA) of rDNA repeats in *Uhrf1* KO, H3K9me3 levels in most repeat elements remained largely unchanged (Figure S7E, Table S6). For example, H3K9me3 levels at long interspersed nuclear element-1 (LINE-1) were unaffected, while major satellite repeats showed only a modest reduction (Figures 5C and S7F), suggesting that H3K9me3 at these repetitive elements are largely maintained independently of UHRF1. CpG methylation levels, however, were reduced at both repeat types^65^. Together with previous findings, our results reveal two distinct classes of H3K9me3 loci in mESCs based on their sensitivity to the loss of UHRF1. Imprinted UHRF1-sensitive loci represent examples of epigenetic domains in which H3K9me3 maintenance is reinforced by DNA methylation, akin to maintenance of H3K9me3 at the inducible reporter locus. At UHRF1-insensitive loci, DNA sequence-dependent or other mechanisms are likely to be required for H3K9me3 maintenance.

### *In silico* protein-protein interaction screening suggests physical contacts that may facilitate the crosstalk between H3K9 and DNA methylation

We next used AlphaFold-Multimer (AF-M) to screen *in silico* for potential interactions among 26 top proteins identified in our genetic analysis (Figure S8A). AF-M predicted several interactions with relatively high interface predicted template modeling (ipTM) scores, including between H3K9 methyltransferases, between HP1 proteins and H3K9 methyltransferases, particularly SUV39H1 and H2, between HUSH subunits (MPP8, TASOR, PPHLN1) and H3K9 methyltransferases, and between DNA and H3K9 methyltransferases (Figure S8A; Table S7). DNMT3L, one of the top hits in the CRISPR screen (Figure 3D), was predicted to interact with G9a and SETDB1, DNMT3A and DNMT3B, HUSH components, and the histone binding proteins HIRA and DAXX (Figure S8A). Although, the interaction of DNMT3L with DNMT3A and DNMT3B is well documented^66,67^, physical interaction with G9a had not been reported (Figures S8A, S8B). AF-M predicted multiple contacts, mainly between a β-sheet localized in the N terminus of G9a ankyrin repeats (aa 702 – 966) and a β-sheet in the ATRX-DNMT3-DNMT3L (ADD) domain of DNMT3L (aa 75 – 207) (Figure S8C). We therefore tested whether purified recombinant glutathione S-transferase (GST)-DNMT3L and G9a interacted in a pulldown assay. A GST-DNMT3L domain spanning the amino-acids from glycine 68 to lysine 220, but not GST alone, pulled down the domain of G9a that extends from aspartate 657 to valine 933 (Figure S8D). Together, these results suggest that DNMT3L-G9a interaction may serve as a direct contact point between the DNA methylation and H3K9me pathways.

To further investigate the role of the predicted interaction between DNMT3L and G9a, in maintaining the inducible heterochromatin system we generated a DNMT3L mutant (*Dnmt3l-3A*) harboring three substitutions (L139A, F140A, I141A) within the ADD domain, which was predicted by AF-M to mediate G9a binding (Figure S9A). HA-tagged DNMT3L WT or DNMT3L 3A were integrated into mESCs and performed co-immunoprecipitation (co-IP). G9a immunoprecipitation in mESCs expressing HA-tagged DNMT3L WT, DNMT3L 3A or untagged control, revealed that G9a specifically pulls down DNMT3L WT, confirmed the physical interaction *in vivo* and highlighted the importance of the mutated residues (Figure S9B). Next, we examined the subcellular localization of DNMT3L WT and DNMT3L 3A by fractionating nuclear extracts into total nuclear, soluble, and chromatin-bound fractions. DNMT3L 3A was significantly reduced in the chromatin-bound fraction and enriched in the soluble nuclear fraction compared to DNMT3L WT, suggesting that the mutations impair its ability to bind chromatin (Figure S9C). Thus, DNMT3L-G9a interaction may play a role in recruiting or stabilizing DNMT3L at chromatin. To assess the functional consequences of disrupting the DNMT3L-G9a interaction, we performed H3K9me3 ChIP-qPCR analysis at the *8x-tetO-eGFP* reporter in mESCs transfected with control siRNAs or siRNAs targeting G9a, DNMT3L, or both. Single knockdown of either G9a or DNMT3L resulted in a reduction of H3K9me3 levels, but the double knockdown did not further reduce H3K9me3, suggesting that G9a and DNMT3L act in the same pathway (Figure S9D). We further knocked down endogenous DNMT3L in mESCs expressing siRNA-resistant DNMT3L WT or DNMT3L 3A and assessed H3K9me3 levels. Cells expressing DNMT3L 3A showed a slight but consistent reduction in H3K9me3 levels compared to DNMT3L WT (Figure S9E). Together these findings support that the DNMT3L-G9a interaction contributes to H3K9me3 maintenance.

## Discussion

**Figure 6.**
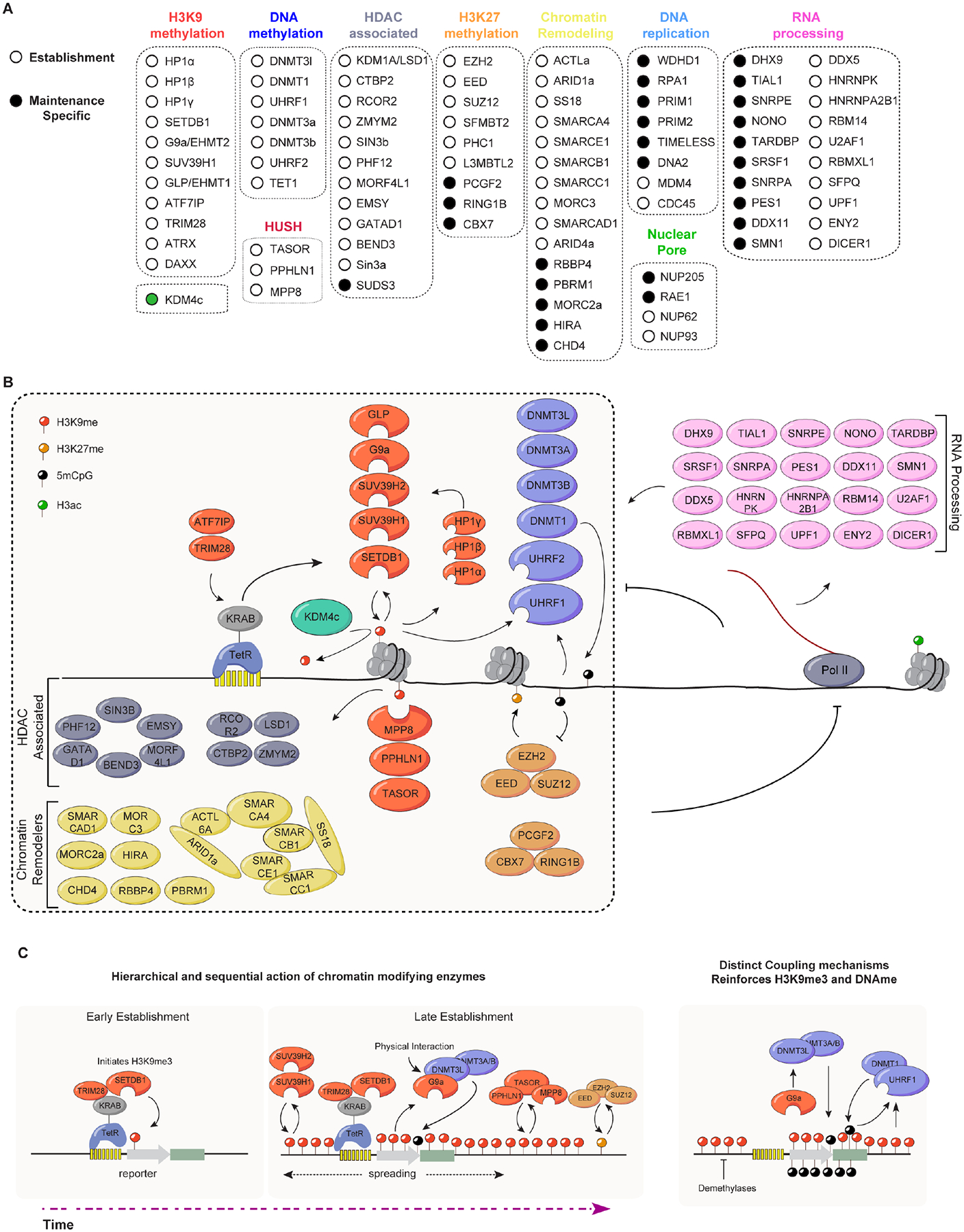
Requirements for the establishment and epigenetic inheritance of H3K9 methylation. **(A)** Representative proteins required for both establishment and maintenance (white circles), maintenance-specific (black circles) and KDM4c (green, silencing enhancer). (B) Schematic highlighting the genetically required molecular activities for H3K9me-dependent silencing, organized into representative functional groups. (C) Sequential recruitment of chromatin-modifying enzymes during heterochromatin establishment. SETDB1 initiates H3K9me3 deposition during early establishment, while SUV39H1/2 and G9a/GLP promote the subsequent accumulation of H3K9me3. G9a directly recruits *de novo* DNA methyltransferases through interaction with DNMT3L at H3K9me-enriched regions, whereas UHRF1 recruits DNMT1 through recognition of H3K9me3 and DNA methylation. Together these pathways reinforce H3K9me maintenance against active demethylation. The opposing activities of demethylases, which vary during differentiation, influence the requirements for heterochromatin maintenance. See also Figures S8–S9.

Our findings provide a comprehensive overview of the pathways that are required for the establishment and epigenetic maintenance of a transiently induced domain of H3K9me3 and gene silencing at a genic locus in mESCs. In addition to identifying requirements for several pathways known to be associated with H3K9me3 heterochromatin, our findings suggest a critical role for 5mC DNA methylation in epigenetic maintenance of the induced H3K9me3 heterochromatin and identify roles for new pathways (Figures 6A–C). We provide evidence that the epigenetic stability of the induced domain of heterochromatin increases as mESCs are differentiated into neuronal progenitor cells, an event that is concomitant with reduced expression of key H3K9me and DNA demethylases and increased DNA methylation at the locus. These observations suggest that developmental downregulation of erasure pathways enhances heterochromatin stability, a principle that may broadly apply to developmental lineage commitment^68^. Based on the distinct requirements for DNA methylation in maintenance of H3K9me3 at the induced heterochromatin domain and endogenous loci, we proposed that mammalian H3K9me3 heterochromatin can be classified into two distinct types. One type represents genic loci, such as imprinted genes, which behave like the induced heterochromatin locus and are maintained through the coupling of H3K9me3 and DNA methylation. A second type represents repetitive genomic regions in which H3K9me3 is maintained independently of DNA methylation by mechanisms that are likely to involve strong input from locus-specific DNA sequence elements. Below we discuss the implication of these findings for the evolution and mechanisms of heterochromatin inheritance.

The formation of heterochromatin induced by TetR-KRAB is associated with the formation of a domain of H3K9me3 and CpG DNA methylation. While previous studies have provided evidence for the coupling of H3K9me and DNA methylation^69^, our findings suggest that this coupling is critical for epigenetic maintenance of DNA sequence-independent H3K9me3. We show that in mESCs, H3K9me3 by itself is not heritable, despite numerous read-write modules present in the methyltransferases themselves or imparted through their association with HP1 proteins. UHRF1 binds to methylated CpG dinucleotides and H3K9me3 through distinct domains, providing a physical and functional link between H3K9me and DNA methylation, and promotes the ubiquitination of H3 on lysines 18 and 23 to promote DNMT1 recruitment for maintenance of DNA methylation^60,61,65,70–74^. In addition to the UHRF1-DNMT1 pathway, we show that H3K9me can also promote *de novo* DNA methylation through a direct interaction between G9a and DNMT3L, reinforcing the coupling of histone and DNA methylation at newly formed heterochromatin domains (Figure 6C). Consistent with this model, previous studies at imprinted loci have shown that the G9a/GLP complex protects DNA methylation independently of its catalytic activity, likely through the recruitment of *de novo* DNA methyltransferases to antagonize TET-mediated demethylation^75 76,77^. Together, our results suggest that in embryonic stem cells two distinct mechanisms couple H3K9me3 and DNA methylation to compensate for the elevated turnover and demethylase activities associated with the pluripotent cell state (Figure 6C). The G9a-DNMT3L interaction provides a continuous recruitment pathway that counters methylation turnover at metastable loci, while UHRF1 is required to ensure DNMT1-dependent methylation maintenance through direct reading of H3K9me3. The requirement for UHRF1 in maintenance of both DNA methylation and H3K9me may be explained by its reported ability to associate with G9a and SUV39H1^78^, creating reciprocal reinforcing positive feedback loops between DNA methylation and H3K9me3^79^. In its requirement for UHRF1 and DNA methylation, the induced heterochromatin at the reporter locus behaves like imprinted genes. However, in contrast to the reporter locus which displays a high loss rate, H3K9me3 and silencing of imprinted loci are highly stable in mESCs. This is likely due to the presence of specific sequence motifs at imprinted genes that are recognized by ZFP57 and other zinc finger proteins that help recruit SETDB1^80,81^. Other H3K9me3 loci, including long interspersed nuclear element-1 (LINE-1) and major satellites are not sensitive to the loss of UHRF1 and DNA methylation, likely to the presence of robust *de novo* establishment pathways^82^.

Our analysis revealed that, in addition to SETDB1, the other H3K9 methyltransferases G9a, GLP, SUV39H1, and SUV39H2, but not SETDB2, are required for the establishment of *de novo* heterochromatin at the ectopic locus. Previous studies have shown that multiple H3K9 methyltransferases (SUV39H1, G9a, GLP, SETDB1) can associate together in a multimeric protein complex, however, whether they functionally cooperate for silencing of a single locus had not been previously investigated^83^. Through temporal analysis of H3K9me establishment, we found that SETDB1 is uniquely required for the initial deposition of H3K9me3, while SUV39H enzymes and G9a/GLP act later to amplify and stabilize heterochromatin domains (Figure 6C). This hierarchical and coordinated model of sequential methyltransferase action is supported by studies at endogenous loci, where distinct methyltransferases contribute in a non-redundant manner^13,84,85^. Although the precise molecular basis remains to be elucidated, likely possibilities include their roles in the transition from H3K9me1 to H3K9me3 or their distinct read-write abilities that may be required for H3K9me spreading.

In addition to components of H3K9me and DNA methylation pathways, we show that heterochromatin establishment and maintenance require the HUSH complex, HDAC-associated proteins, chromatin remodeling proteins (mainly components of the BAF complex), nuclear pore complex (NPC) subunits and Polycomb group proteins (Figures 6A and 6B). Despite evidence on the role of these proteins in heterochromatin formation and silencing, the molecular mechanisms underlying their functions in heterochromatin establishment and maintenance are not understood^23,48–51,86–92^. The HUSH complex is required for initiation of H3K9me and silencing of LINE-1 retrotransposons, endogenous retroviruses, and long intronless transgenes^82,86–88^. Our findings indicate that the HUSH complex also acts downstream of H3K9me since H3K9me at our reporter locus is initiated by TetR-KRAB binding. Moreover, previous studies in *S. pombe* showed that Rae1 and NUP205 (NPC subunits) are enriched in Swi6/HP1 heterochromatin fraction, and NUP93 is required for clustering of heterochromatic foci and epigenetic inheritance of heterochromatin^93^(Figure 6A). Our data suggests that their role(s) in heterochromatin formation and silencing may be conserved in mammalian cells.

The strong requirement for Polycomb proteins in establishment and maintenance of an H3K9me-dependent silencing is notable. Although co-enrichment of H3K9me and H3K27me has been previously reported on some LINE-1 retrotransposons, and it has also been proposed that DNMTs recognize and target PRC-modified regions^44–47^, the functional importance of these interactions in silencing has remained unclear. Our findings suggest that PRC1/2 complexes contribute to maturation of H3K9me heterochromatic domains, for example, by binding to CpG islands prior to their methylation by DNMTs.

Our identification of replisome proteins as maintenance-specific factors is consistent with the central role of the replication machinery in parental histone transfer and epigenetic inheritance^94^. For example, replication protein A (RPA) (Figure 6A) binds single-stranded DNA during DNA replication and has a role in replication-coupled nucleosome assembly^95^, the WD Repeat and HMG-Box DNA Binding Protein 1 (WDHD1) has been implicated in the formation of pericentric heterochromatin in mammalian cells^96^, and components of the replication fork protection complex, including Mrc1/CLASPIN and Swi1/TIMELESS are required for parental histone recycling and heterochromatin maintenance^97–100^. Additionally, a previous study identified mutations in replisome proteins that result in defective heterochromatin maintenance in fission yeast *S. pombe*^29^, several of which were also revealed by our screen. Together with previous studies, our results suggest that the DNA replication machinery plays a conserved role in epigenetic inheritance of heterochromatin.

Components of the RNAi and other RNA processing pathways have been identified with critical roles in the establishment and maintenance of silencing in organisms ranging from fission yeast to human^4,21^ Although the RNAi pathway does not seem to play a direct role in heterochromatin formation in mammalian somatic cells, our results suggest that other RNA processing pathways that likely regulate nascent RNA metabolism are critical for the stability of heterochromatin in mESCs. Among these pathways, we note roles for RNA or RNA-DNA helicases (DHX9, DDX5, and DDX11), SFPQ/NONO paraspeckle components, and multiple splicing regulators.

Overall, our findings provide insight into the main principles and conserved pathways that may regulate the epigenetic stability of heterochromatin in evolutionarily distant organisms. First, epigenetic stability of heterochromatin appears to be regulated by anti-silencing or erasure factors. In the fission yeast *S. pombe*, DNA sequence-independent inheritance of heterochromatin is regulated by an anti-silencing protein that promotes H3K9 demethylation^7,8^. In mammalian cells, the stability of heterochromatin appears to be similarly affected by H3K9me and DNA 5mC demethylases whose expression is reduced upon differentiation of mESCs leading to increased epigenetic stability of newly acquired heterochromatin. Second, in both fission yeast and mouse cells, heterochromatin domains can be stably maintained through the coupling of H3K9me read-write with another positive feedback mechanism, small RNA amplification in *S. pombe* and 5mC read-write in mESCs. Third, in the absence of reinforcement by another positive feedback mechanism, epigenetic inheritance of H3K9me is achieved by input from context- and DNA sequence-specific factors such as site-specific DNA binding proteins in both yeast and mESCs. Finally, this study provides a comprehensive view of the requirements for heterochromatin establishment and maintenance that will serve as a resource for future studies.

### Limitations of the Study

Our study examines the requirements for the establishment and maintenance of an induced domain of H3K9me3 heterochromatin at a single euchromatic locus. Although we have examined some of the requirements for this induced domain of heterochromatin, such as the coupling of H3K9me3 and DNA methylation, and the requirement for multiple H3K9 methyltransferases, and our conclusions are also consistent with prior studies, we cannot rule out the effect of local chromatin structure on the behavior of our reporter gene. DNA sequence has been shown to affect the inheritance of induced H3K9me3 heterochromatin in fission yeast and H3K27me3 heterochromatin in human cells^9,10,101^. Additional experiments are required to explore how DNA sequence at the integration site may affect H3K9me3 heterochromatin inheritance in mammalian cells.

### Resource Availability

#### Lead Contact

Further information and requests for reagents should be directed to and will be fulfilled by the Lead Contact, Danesh Moazed (danesh@hms.harvard.edu). The materials generated in this study would be shared without restriction.

#### Materials Availability

Resources and materials generated in this study are available upon request and the request should be directed to lead contact Danesh Moazed.

#### Data and Code Availability

- The raw membrane and microscopy images were deposited at Mendeley Data at (https://data.mendeley.com/datasets/btdkb5nnnw/1, DOI: 10.17632/btdkb5nnnw.1) and are publicly available on the date of publication. AlphaFold-Multimer-predicted structures are deposited on ModelArchive under the accession number ma-dm-mmk9 and are publicly available on the date of publication. CRISRP-Cas9 screen and genome-wide datasets are deposited in the Gene Expression Omnibus (GEO) under the accession number GSE212153. Accession numbers for datasets are also listed in the Key Resources Table.
- This paper does not report original code.
- Any additional information required to reanalyze the data reported in this paper is available from the lead contact upon request.

## STAR★Methods

### Experimental Model and Study Participant Details

#### Cell Culture

E14TG2a mES cells (ATCC, #CRL-1821) were cultured on 0.1% gelatin-coated (Sigma, #G1890) plates in Dulbecco’s modified Eagle’s medium (DMEM) supplemented with 15% heat-inactivated fetal bovine serum (FBS) (ThermoFisher, #10437), 1% MEM non-essential amino acid (ThermoFisher, #11140), 2 mM Glutamine (ThermoFisher, #25030081), 100 μM β-mercaptoethanol (Sigma, #63689), 10 U/ml penicillin/streptomycin (ThermoFisher, #15140), 1,000 U/ml LIF (StemCell Technologies, #78056) at 37 °C with 5% CO2. 293FT cells (ThermoFisher, #R70007) used for the production of lentiviruses were maintained in DMEM with 10% heat-inactivated FBS, 2 mM glutamine and 100 U/ml penicillin/streptomycin following standard culture conditions. NPCs are cultured in N2B27 medium (1:1, Neurobasal:DMEM-F12, supplemented with N2 and B27, ThermoFisher) in the presence of 10 ng/ml bFGF (Peprotech) and 10 ng/ml EGF (Stem Cell Technologies). NPCs were dissociated gently with accutase (Invitrogen) for passaging and cultured in 5 μg/ml doxycycline when indicated.

### Method Details

#### Generation of the reporter mESCs line

The *8x-tetO-eGFP* reporter construct was generated as follows: ~800bp homology arms (flanking both sides at the integration site, found at position +37bp in NM011701 (*Vimentin* gene)) were PCR amplified from gblocks gene fragments synthesized at Integrated DNA Technologies (IDT). PCR amplified *8xtetO-EF1a* promoter-eGFP cassette from iDuet101a (Addgene, #17629) was cloned with Gibson assembly in frame with the SV40pA sequences that were PCR amplified from lentiCRIPSRv2 (Addgene, #52961). Gibson cloning was subsequently used to simultaneously encompass digested *8xtetO-EF1a* promoter-eGFP-SV40pA cassette in frame with the homology arms and the whole insert was cloned into the pSMART-HCKAN (Lucigen) vector, assembling the donor plasmid. To integrate the reporter gene in the genome, homology-dependent recombination mediated by CRISPR-Cas9 genome editing was induced via a spCas9/sgRNA ribonucleoprotein (RNP) complex reconstituted *in vitro* before insertion into the cells. sgRNA (5′-TGTGAATCGTAGGAGCGCTG- 3′) was synthesized via *in vitro* GeneArt^™^ Precision gRNA Synthesis Kit (ThermoFisher, A29377) following manufacturer’s instructions. SpCas9 protein was purified by the Initiative for Genome Editing and Neurodegeneration Core in the Department of Cell Biology at Harvard Medical School. Donor plasmid, and spCas9/sgRNA RNP were delivered to cells by electroporation with Neon transfection system (ThermoFisher, MPK1025). Single eGFP^+^ cells were FACS sorted and seeded in 96-well plates. Single clones were picked, expanded, and screened for insertion by PCR. Validation of positive clones was performed by sanger sequencing.

*8xtetO-EF1a-eGFP* reporter mESC line was further engineered to harbor TetR-3xFlag-KRAB protein or TetR-3xFlag inserted at the *Rosa26* locus. The constructs for these lines were generated as follows: ~650–750bp homology arms (flanking both sides at the integration site, found at position chr6:113,076,053 (GRCm38/mm10) (Gt(ROSA)26Sor) were PCR amplified from gblocks gene fragments synthesized at IDT. First, we generated PCR amplified EF1a promoter and TetR-NLS signal cassette from pLV-tTRKRAB-red (Addgene, #12250). 3xFlag-KRAB was PCR amplified from the same vector with a forward primer including the sequence encoding 3xFlag and SV40pA amplified from lentiCRIPSRv2. The inserts were cloned with Gibson assembly in frame with a neomycin-HSV tk pA cassette and the homology arms (HomL or HomR). The whole cassette (HomL-EF1a-TetR-3xFlag-KRAB-SV40pA-NeoR-HomR) with or without KRAB was cloned into the pSMART-HCKAN (Lucigen) vector, assembling the donor plasmid. To integrate the above cassettes in the genome the same method was used as above using sgRNA sequence (5′-ACTGGAGTTGCAGATCACGA-3′) that targets intron1 of the *Rosa26* locus. Single cells were FACS sorted and selected for neomycin resistance. Single clones were picked, expanded, and screened for insertion by PCR. Validation of positive clones was performed by sanger sequencing and western blot.

#### EpiChromo Library generation

Single guide RNAs (sgRNAs) targeting 1160 genes including all known chromatin and epigenetic regulators, DNA replication factors, nuclear periphery factors, RNA processing factors, and proteins identified in proteomic analyses of heterochromatin composition were designed to generate a pooled library, named EpiChromo (Table S1). The optimized sgRNA design strategy and rule set scoring (Azimuth 2.0) to determine editing activity scores described earlier^102^ (http://www.broadinstitute.org/rnai/public/analysis-tools/sgrna-design) were used as the basis for the design of EpiChromo sgRNAs. Top-5 ranked sgRNAs targeting the coding domains of each gene after on-target and off-target analysis were picked for library construction. We excluded sgRNAs with a BsmBI recognition site in their sequence to allow subcloning into the lentiCRISPRv2 plasmid after BsmB1 digestion. We included 150 control sgRNAs that do not target the mouse genome in the EpiChromo library resulting in a total of 5,950 sgRNAs (Table S2). EpiChromo library is also available at the Gene Expression Omnibus (GEO) repository under accession number GSE212153.

Library generation was performed as described earlier^102,103^. Briefly, to each sgRNA sequence, BsmBI (Esp3l) cleavage sites sequences (underlined) along with the appropriate BsmBI recognition sequences (CGTCTCACACCG (sgRNA, 20 nt) GTTTCGAGACG) were added for cloning into the lentiCRISPRv2 (Addgene, #52961) sgRNA and spCas9 expression system. Additional sequences were added for the amplification of the library (italics) and the final oligonucleotide sequence was thus: 5′-(*AGGCACTTGCTCGTACGACG*) CGTCTCACACCG (sgRNA, 20 nt) GTTTCGAGACG (*ATGTGGGCCCGGCACCTTAA*)-3′. A pool of 5,950 oligonucleotides was synthesized (Twist Biosciences). A primer set annealing to the amplification sequences was used at a final concentration of 0.5 μM to amplify the pool of oligonucleotides using 12.5 μL 2 × NEBnext PCR master mix (New England BioLabs) and 3 μL of oligonucleotide pool (~3 ng), at a final volume of 25 μL. PCR cycling conditions were 15 s at 98 °C, 20 s at 60 °C, 30 s at 72 °C, for 18 cycles. The resulting amplicons were PCR-purified (NucleoSpin^®^ Extract II, Clontech). To clone the pool of oligonucleotides in the lentiCRISPRv2 vector, we set up Golden Gate assembly reactions at a ratio of 25:1 with 500 ng of vector in reaction buffer (Tango buffer (1x), 0.5 μL DTT (100 mM), 0.5 μL ATP (100 mM), 1 μL (BsmBI) Esp3l (Thermo Fisher Scientific), 1 μL T7 ligase) at 50 μL final volume. Golden Assembly reaction was performed using the following cycling conditions: 5 min at 37 °C, 5 min at 20 °C, for 100 cycles. Golden Gate reaction products were isopropanol precipitated using 1 μL of GlycoBlue (15 mg/ml), 4 μL NaCl (5M) and 50 μL isopropanol. After incubation at room temperature for 15 min, we pelleted the library by centrifugation at 13,000 *g* for 20 min, washed the pellet twice with ice-cold 70% ethanol, and centrifuged after each wash at 13,000 *g* for 5 min. After drying, the pellet was resuspended in nuclease-free water. We then performed electroporation using the *E. coli* cells (Endura electrocompetent cells, Lucigen) following the manufacturer’s instructions transforming 90 ng of library DNA into 25 μL of electrocompetent cells for each electroporation reaction for a total of 6 reactions and the cells were pooled and plated on 6 × LB agar plates (245-mm square bioassay dish, 100 μg/ml carbenicillin) and were grown at 30 °C for 20–24 h. Colonies were scraped and plasmid DNA (pDNA) of the sgRNA library was prepared (HiSpeed Plasmid Maxi, Qiagen). Deep-sequencing confirmed that all sgRNAs were well represented in the EpiChromo library. The relative difference in the abundance of sgRNAs in the library at the 10th and 90th percentiles was ~3.6-fold. sgRNA sequences for the EpiChromo library are presented in Table S2.

To generate the virus used for the CRISPR-Cas9 screens, 293FT cells were plated on 150-mm plates and cultured to 80% confluency in order to perform transfection with the pDNA library. Transfection was performed using Lipofectamine 2000 (ThermoFisher) according to the manufacturer’s directions. Briefly, for each transfection in one 150-mm plate, 135 μL of lipofectamine 2000 was added in 4.5 ml of Opti-MEM (Corning) and incubated at room temperature for 5 min. A second solution contained 10.2 μg pMD2.G (Addgene, #12259), 15.6 μg psPAX2 (Addgene, #12260), and 20.4 μg of the library pDNA in 4.5 ml of Opti-MEM. The two solutions were combined and incubated at room temperature for 15–20 min. During this incubation period, the medium on the 293FT cells was changed with 15 ml of fresh prewarmed medium. At the end of the incubation period the transfection mixture was added dropwise to the cells, and then cells were incubated at 37 °C for about 10–12 h after which the transfection medium was removed and replaced with the fresh prewarmed medium. The virus was harvested ~56 h post-transfection. After clarification of the medium to remove dead cells and debris by centrifugation and passage through 0.45 μm filters, viral particles were collected by using PEG-it Virus precipitation solution following manufacturer's instructions. Finally, virus was resuspended in mESCs culturing medium and titration was performed using mESCs.

#### Establishment and Maintenance CRISPR-Cas9 screens

The screens were performed generally as described previously with some modifications^102,103^. Each screen was performed in duplicate. Briefly, for each replicate we used 6 × 10^7^ mESCs carrying the inducible silencing system cultured in medium with 10 μg/ml doxycycline to prevent silencing of the reporter gene. Cells were transduced with the EpiChromo pooled sgRNA library of virus at a multiplicity of infection (MOI) of approximately 0.1 in the presence of polybrene at a final concentration of 5 μg/ml. This results in 6 × 10^6^ transduced mESCs, which is sufficient for the integration of each individual sgRNA into ~1,000 unique cells. Two days post-transduction, doxycycline (Dox) was removed from the medium to induce silencing of the reporter gene and at the same time 0.25 μg/ml of puromycin was added to the medium to eliminate non-transduced mESCs. The cells were cultured at the minimum number of 6 × 10^6^ cells for each replicate throughout the course of screening in order to ensure coverage of the sgRNAs library in the cell population at ~1,000 per sgRNA. For the establishment screen, at the end of the establishment period (10 days in −Dox medium), FACS was used to isolate ~ 3 × 10^6^ eGFP^+^ cells per replicate. Equal numbers of unsorted population of transduced cells were used as controls for comparison. For the maintenance screen, at the end of the establishment period, FACS was used to isolate ~22 × 10^6^ eGFP^−^ cells per replicate, which were seeded and further cultured. After 16 h of sorting and seeding, the cells were cultured for 3 days in medium with 10 μg/ml Dox to release TetR-KRAB from the 8x-tetO sites and assess how the mutant population of cells perform during the maintenance phase of silencing. This 3-day time point was chosen to allow for approximately 5–6 cell divisions, a period sufficient to uncover defects in the maintenance of H3K9me3-mediated silencing while minimizing background noise from spontaneous loss of the silent state. Notably, this window captures the transition from sequence-dependent to sequence-independent silencing. The cells were cultured at the minimum number of 6 × 10^6^ cells during the maintenance phase in order to ensure coverage of the sgRNAs library in the cell population at ~1,000 per sgRNA. At the end of the maintenance period, FACS was used to isolate ~3 × 10^6^ eGFP^+^ cells per replicate and again equal numbers of unsorted cells were used as controls for comparison. From FACS sorted and unsorted cells for both screens, cell pellets were treated with proteinase K and DNAse-free RNase A in lysis buffer (50 mM KCl, 10 mM Tris-HCl (pH8.3), 2.5 mM MgCl2-6-H2O, 0.45% NP-40, 0.45% Tween-20) and genomic DNA (gDNA) was initially purified using phenol/chloroform/isoamyl alcohol and then ethanol precipitated. PCR of gDNA was performed to attach sequencing adaptors and barcode samples. gDNA of each sample was divided into multiple 50 μL reactions containing a maximum of 2.5 μg gDNA per reaction. For each PCR reaction we used: 0.75 μL of ExTaq DNA polymerase (Takara), 5 μL of (10x) ExTaq buffer, 4 μL of dNTPs provided with the enzyme, 2.5 μL of P5 stagger primer mix (stock at 10 μM) and 2.5 μL of P7 uniquely barcoded primer (stock at 10 μM). PCR cycling conditions were an initial 3 min at 95 °C; followed by 30 s at 95 °C, 30 s at 53 °C, 30 s at 72 °C, for 23 cycles; and a final 10 min extension at 72 °C. PCR products for each sample were pooled, electrophoresed in an agarose gel, purified (NucleoSpin^®^ Extract II) and sequenced on an Illumina NextSeq 500.

#### Screen analysis

The sgRNA counts and abundance were analyzed as described^103^. Guide sequences were extracted from the raw sequencing reads with a modified version of the Python script “count_spacers.py”^103^ using a “CGAAACACC” search prefix, and a counts matrix was generated in each sample. Next, counts files of eGFP^+^ sorted cells and of unsorted population of cells subject to comparison were input into MAGeCK algorithm (version 0.5.9.4)^36^ and log_2_ fold changes (LFCs) and *P*-values (negative binomial distribution) were calculated for each sgRNA using the ‘mageck test −k’ command with default settings. MAGeCK uses a modified robust ranking aggregation (α-RRA) algorithm to calculate gene level *P*-values and the Benjamini-Hochberg procedure to calculate gene level false discovery rate FDR values^36,104^. Gene level LFC were calculated by averaging LFCs of all 5 sgRNAs targeting a given gene. The results of this analysis are in Tables S3 and S4. The following criteria were used to identify candidate silencing genes with more confidence: i) An FDR value of < 0.1 as a cutoff, ii) at least 3 out of 5 effective (positively selected) sgRNAs, and iii) average of LFCs of the effective sgRNAs (alphamean) higher than 0.585 for the maintenance screen. Using these criteria, we identified 79 and 121 factors affecting silencing during establishment and maintenance, respectively.

Among the 121 factors identified in the maintenance screen, 38 were also found in the establishment screen, and the rest 83 were found to affect silencing only during maintenance based on the above criteria. To increase confidence that we identify maintenance-specific genes, we excluded 19 additional genes (out of 83), which had an effect during establishment at a lower FDR cut-off (FDR < 0.2) than the cut-off originally used (FDR < 0.1). Z-scores used in figures were calculated for each sgRNA, Z = (x-m)/s, where x is the LFC for a sgRNA, m is the mean LFC of all sgRNAs, s is the standard deviation of all sgRNAs, and mean Z-score was calculated by averaging the Z-scores of all 5 sgRNAs per gene. CRISPR-Cas9 screen data (fastq and tables) are available at the Gene Expression Omnibus (GEO) repository under accession number GSE212153.

To compare the factors identified in our screen with reported heterochromatin-associated proteins, we integrated proteomic data from five independent studies^11,37–40^ that employed distinct strategies, including isolation of pericentromeric heterochromatin using LNA probes^11^, chromatin fractionation based on sonication resistance and compaction^37^, proximity biotinylation using engineered chromatin readers^38^ or antibody-directed TurboID labeling of H3K9me3-enriched regions^39^, and sucrose gradient purification of native pericentromeric heterochromatin^40^. Together, these approaches identified proteins interacting with H3K9me3-marked chromatin, or enriched in constitutive heterochromatin domains, characterizing mammalian heterochromatin composition (Figures S2D, S5G, Tables S3 and S4).

#### Functional enrichment and network analysis

Functional enrichment analysis of establishment factors for Figure S2 was performed using g:Profiler (version e106_eg53_p16_65fcd97), with EpiChromo library targeted genes as background list of genes, and Benjamini-Hochberg multiple testing correction method applying significance threshold of 0.05 to retrieve the GO_Biological process (GO_BP) and the GO_Cellular Component (GO_CC) terms enriched^105^.

We performed network analysis using the STRING (version 11.5) database to identify functional interactions (high confidence ≥ 0.85) between the candidate silencing genes^41^. Markov clustering in STRING was performed with an inflation value of 2.5 and array source experiments with no cut off. We used *Mus Musculus* gene symbols and analyzed interactions from all available sources provided by that tool. We performed STRING-based analysis, clustering, and visualization of the network of gene interactions in Cytoscape (version 3.9.0) using a circular layout^106^. Edge weights represent confidence score between gene interaction, and nodes and node weights illustrate genes and log2FC respectively. Colored edges represent clustered gene interactions calculated by Markov clustering. Protein annotations from STRING database were used in Tables S3 and S4.

#### Screen hit validation

SgRNAs used for the assessment of screen hits were cloned into lentiCRISPRv2 which also encodes the spCas9 gene as described^103^. Individual sgRNAs sequences used to target the indicated genes were the ones used for the pooled CRISRP screens. 4 × 10^4^ mESCs seeded in each well of 96-well plates carrying the inducible silencing system cultured in medium without doxycycline for 6 days prior to infection to induce silencing were individually transduced with lentiviruses carrying one sgRNA per gene in the presence of polybrene at a final concentration of 5 μg/ml. Three days post-infection transduced cells were selected with 1 μg/ml puromycin for three more days in medium without doxycycline. At the end of this selection period, half of the cells for each condition were cultured in the same medium without doxycycline and the other half in the presence of 10 μg/ml doxycycline to assess maintenance. Cells were cultured for three days before assessment of silencing by FACS analysis and the % of eGFP^+^ cells (derepressed cells) was assessed.

#### Flow cytometry

Cells were gently dissociated into single-cell suspension using Accutase (Invitrogen) and resuspended in PBS+1% FBS (FACS medium) and filtered through FACS tubes (Corning^™^ #352235) with cell strainer cap. FACS was performed using a BD FACSAria II and FACSAria II SORP high speed cell sorters. For silencing assays and screen hit validation, samples were run on a BD FACSCalibur system and the iQue Screener PLUS (IntelliCyt) system. Data were analyzed using FlowJo (version 10.7.2). Single cells were determined by analyzing singlets by comparing cell size (FSC-A) and cell granularity (SSC-A). eGFP gating (eGFP^+^ and eGFP^−^) was determined from the comparison of the distribution of eGFP values from wild-type and *8x-tetO-eGFP* mESCs.

#### Generation of KDM4c and DNMT3L WT/3A mESCs lines

Mouse KDM4c cDNA was obtained from horizon discovery (Clone ID: 5357211). PCR-amplified with Gibson overhangs and a 3xHA tag for insertion into the Fuw-dCas9-Dnmt3a-P2A-Puro backbone. The vector was digested with EcoRI and BamHI, then assembled with the KDM4c PCR product using Gibson assembly, replacing the dCas9-Dnmt3a sequence with KDM4c-3xHA. DNMT3L was amplified from mESC cDNA with Gibson overhangs and a 3xHA tag, then cloned into the same backbone (digested with AscI and EcoRI) to replace dCas9-Dnmt3a. The original Fuw-dCas9-Dnmt3a-P2A-Puro plasmid was generated through Gibson assembly of three components: (1) an HA-P2A-Puro fragment amplified from lentiCRISPR V2 (Addgene #52961), (2) a WPRE-containing fragment from Fuw-dCas9-Dnmt3a-P2A-tagBFP (Addgene #2527), and (3) the EcoRI/PmeI-digested Fuw-dCas9-Dnmt3a-P2A-tagBFP backbone. For the DNMT3L 3A mutant (L139A, F140A, I141A in the ADD domain), we performed site-directed mutagenesis using primers listed in Table S8. RNAi-resistant versions of both DNMT3L WT and 3A were similarly generated using mutagenesis primers found in Table S8. The siRNA target sequence was mutated from 5’- CGACGGAGCATTGAAGACA-3’ to 5’-AGAAGATCCATCGAAGACA-3’. Full Plasmids were sequenced using nanopore sequencing (Quintara Bio). For integrating the plasmids expressing KDM4c-3xHA and DNMT3L-3xHA different versions, mESCs were transduced with the appropriate lentiviruses produced from the Fuw-KDM4c-3xHA-P2A-Puro or Fuw-DNMT3L WT/3A-3xHA-P2A-Puro (including RNAi resistant variants). Following transduction, cells were selected with puromycin (1 μg/mL) for the specified duration prior to experiments. This ensured stable integration of the HA-tagged constructs (KDM4c or DNMT3L WT/3A mutant) while maintaining RNAi resistance where applicable.

#### Neuronal Progenitor Cell differentiation

Differentiation of mESCs to neural progenitor cells (NPCs) was performed as previously described with some modifications^107^. mESCs were single cell dissociated and aggregated in 24 well Aggrewell 400 plates (Stemcell, # 34425) coated with anti-adherence rinsing solution (Stemcell, #07010) at a density of 16 cells per microwell in cell aggregate (CA) medium. Four days after the onset of embryonic body (EB) formation, we added 2 μM retinoic acid (Sigma R-2625). After dissociation of EBs, cells were seeded on 0.1% gelatin-coated plates and NPCs are cultured in N2B27 medium (1:1, Neurobasal:DMEM-F12, supplemented with N2 and B27, ThermoFisher) in the presence of 10 ng/ml bFGF (Peprotech) and 10 ng/ml EGF (StemCell Technologies)^108^. NPCs were dissociated gently with accutase (Invitrogen) for passaging and cultured in 5 μg/ml doxycycline when indicated. For KDM4c overexpression studies, mESCs were transduced with lentivirus produced from the Fuw-KDM4c-3xHA-P2A-Puro vector following the outline in Figure 4G. Cells were cultured in 2i/LIF medium with titrated PD032590 (t2il) to maintain similar DNAme levels to Serum LIF culture while eliminating differentiated cells during puromycin selection (1μg/ml). For 250 mL culture medium, we combine 125 mL each of DMEM/F12 (Invitrogen) and neurobasal medium (Invitrogen) supplemented with 0.5x N2 supplement (5 mL, Thermo Fisher Scientific), 0.5x B27 supplement (10 mL, Thermo Fisher Scientific), GlutaMAX (2.5 mL, Thermo Fisher Scientific), nonessential amino acids (2.5 mL, Thermo Fisher Scientific), 0.1 mM β-mercaptoethanol (Sigma), 10 ng/ml LIF (Stemcell, #78056), 3 μM Chir99021 (MedChemExpress, HY-10182G), 0.2 μM PD0325901 (MedChemExpress, HY-10254), and 1% FBS.

#### Imaging

Epifluorescence and phase contrast images were taken on a Nikon Eclipse Ts2 equipped with the following objective 4x/0.13 numerical aperture (NA) air, 10x/0.25 NA air, 20x/0.4 NA air, 40x/0.55 NA. All images were acquired with a photometrics 2.8megapixel CoolSNAP Myo camera through a Nikon NIS Elements imaging software (Version 4.51). All imaging experiments were repeated at least twice representative images are shown.

#### Immunofluorescence staining

Cells were fixed with 4% paraformaldehyde (PFA) in 1xDPBS for 20 min at room temperature, washed in wash buffer (0.1% Triton X-100, 2% BSA in 1xDPBS) for 15 min and permeabilized with 0.5% Triton X-100 in 2% BSA PBS for 30 min. Samples were washed in wash buffer for 5 min. 1×10^5 cells where stained with Primary antibodies were diluted in wash buffer α-HA (Mouse, Thermo, 26483, 0.5ng/μl) and α-Sox2 (Goat, Biotechne, AF2018, 0.5ng/μl). Cells where incubated with primary antibody for 1h at room temperature and washed twice for 15 min in wash buffer and incubated with fluorescent-dye-conjugated Donkey secondary antibodies (AF-594, AF- 647 Invitrogen) diluted in blocking buffer (wash buffer + 5% Donkey serum (1:300 dilution) for 1 h at room temperature or overnight at 4 C. Samples were washed twice with wash buffer.

#### Generation of knockout mESCs line

To generate *Uhrf1* clonal knockout lines in mESCs carrying the inducible heterochromatin system, CRISPR-Cas9 method was applied. sgRNAs were cloned into the lentiCRISPRv2 vector as described^103^ (Table S8). Cells were transiently transfected with the plasmid carrying the appropriate sgRNA using Lipofectamine 3000 (ThermoFisher) following manufacturer’s directions. Lipofectamine 3000 and plasmid were incubated with the cells for 3 h and then replaced with fresh medium. One day after transfection, cells were selected with 1 μg/ml puromycin for three days and then single cells were FACS isolated and seeded in 96-well plates and cultured without puromycin for the rest of clonal expansion. Single clones were expanded and screened by genotyping and MiSeq (Illumina). Knockout clones were validated by western blot.

To generate knockout lines of TetR-3xFlag-KRAB, 2 sgRNAs were used against the *tetR* sequence specifically. sgRNAs were cloned into the lentiCRISPRv2 vector and lentiviruses were prepared. For establishment, cells cultured in doxycycline were transduced simultaneously with the two viruses in the presence of polybrene at a final concentration of 5 μg/ml. Two days post-infection, cells were selected with 1 μg/ml puromycin and cultured in medium without doxycycline to induce silencing. Ten days later, the cells were assessed for silencing by FACS analysis. For maintenance, cells were cultured without doxycycline for 7 days to induce silencing and then were transduced simultaneously with the two viruses in the presence of polybrene. Two days post-infection, selection with puromycin started and one day later doxycycline was added back to the medium. Four days after adding back doxycycline, cells were assessed for maintenance of silencing. Successful knockout was validated with western blot for 3xFlag, and for the maintenance assay was performed two days after puromycin selection (Day 1 of the maintenance assay).

#### Chromatin immunoprecipitation (ChIP) and quantitative real time PCR (qPCR)

The ChIP assays were performed as described previously^109^. Briefly, to crosslink chromatin, cells were treated with 1% formaldehyde for 10 min at room temperature. Crosslinking was quenched by the addition of glycine to a final concentration of 125 mM for 5 min. The cells were washed twice with ice-cold 1x PBS, scraped into 10 ml of ice-cold PBS, and pelleted by centrifugation at 1000 *g* for 5 min at 4°C. Nuclei were prepared by suspension of cell pellets in a hypotonic buffer (20 mM HEPES pH 7.9, 10 mM KCl, 0.5 mM spermidine, 0.1% Triton X-100, 20% Glycerol, and protease inhibitors), and incubated for 15 min on ice to allow swelling of cells. We then performed dounce homogenization and nuclei were pelleted by centrifugation at 1000 *g* for 5 min at 4°C. The nuclei were lysed in sonication buffer (10 mM Tris-HCl pH 7.5, 1 mM EDTA pH 8.0, 0.1% SDS, and protease inhibitors) and chromatin was sheared with the Covaris E220 Focused-Ultrasonicator (140 watt peak incident, 5% duty factor, 200 cycles/burst, 330 secs sonication time for histone modifications and 420 seconds for chromatin factors) to a fragment distribution of 200 – 500 bp. Sheared chromatin was diluted with 2x dilution buffer (50 mM HEPES pH 7.9, 280 mM NaCl, 1 mM EDTA, 2% Triton X-100, 0.2% Na-deoxycholate, 0.1% SDS, and protease inhibitors). Chromatin samples were incubated with specific antibodies in the final ChIP buffer (25 mM HEPES pH 7.9, 140 mM NaCl, 1 mM EDTA, 1% Triton X-100, 0.1% Na-deoxycholate, 0.1% SDS, and protease inhibitors) overnight at 4 °C. The protein–DNA complexes were immobilized on pre-washed in ChIP buffer protein A or G dynabeads. For each ChIP, the following antibodies were used with Invitrogen dynabeads: 3 μg of anti-H3K9me3 (Abcam 8898) with protein A, 10 μg of anti-Flag (Sigma F1804) with protein G, 3 μg of anti-H3K27me3 (Millipore, #17622) with protein A, 3 μg of anti-H3K4me3 (Sigma 04–745) with protein A, 5 μg anti-SETDB1 (Proteintech Group, 11231–1-AP) with protein G, 5 μg anti-SUV39H1 (Diagenode, 15410368) with protein G, 5 μg anti-G9a (Cell Signal Technology 3306S) with protein G and 5 μg anti-SUZ12 (Cell Signaling 3737S) with protein G. For histone modification ChIPs, we used 3 million cells per ChIP, while for anti-Flag, anti-SETDB1, anti-SUV39H1, anti-G9a, and anti-SUZ12 ChIPs, we used 30 million cells per ChIP. The bound fractions were washed twice with ChIP buffer, twice with high-salt wash buffer (50 mM HEPES pH 7.9, 500 mM NaCl, 1 mM EDTA, 1% Triton X-100, 0.1% Na-deoxycholate, 0.1% SDS, and protease inhibitors), twice with low-salt wash buffer (20 mM HEPES, 250 mM LiCl, 1 mM EDTA, 0.5% NP-40, 0.5% Na-deoxycholate, and protease inhibitors), and twice with TE buffer (10 mM Tris-HCl pH 8.0, 1 mM EDTA). Elution was carried out with 400 μL elution buffer (50 mM Tris-HCl pH 8.0, 100 mM NaHCO3, 1 mM EDTA, and 1% SDS) at 65 °C for 20 min and then 21 μL of NaCl (4M) was added to each eluate and crosslinks were reversed at 65 °C for 10–12 h. After DNase-free RNase A and then proteinase K digestions, DNA samples were purified using phenol/chloroform/isoamyl alcohol and ethanol precipitated with 20 μg glycogen carrier. The precipitated DNA samples were either analyzed by qPCR on Applied Biosystems qPCR instrument in the presence of SYBR green with the primer sequences listed in Table S8 using the ΔCT method, or prepared for DNA high throughput sequencing. Reported values are percent of input or occupancy relative to percent input of a negative control region. qPCR quantification and statistical analysis was performed with at least three biological replicates per condition. Data in all figures are presented as mean values +/− S.D. (Standard Deviation).

#### siRNA-mediated Knockdown

For siRNA-mediated knockdown, cells were transfected with 50 nM siRNA using Lipofectamine RNAiMAX (Thermo Fisher Scientific 13778150) following the manufacturer’s instructions. All siRNAs were synthesized by Dharmacon (see Table S8 for siRNA sequences). Knockdown efficiency was assessed 24 or 72 hours post-transfection by Western blot. Non-targeting siRNA was included in all experiments to control for off-target effects.

#### Protein analysis

Protein samples were loaded on 4–20% gradient TGX Gels (Biorad). SDS-PAGE was performed to separate proteins at 100 Volts for the appropriate amount of time, and proteins were then transferred to a nitrocellulose membrane (Millipore). The membranes were blocked in 5% non-fat dry milk in PBS with 0.2% Tween-20, and sequentially incubated in 1% non-fat dry milk with primary antibodies and HRP-conjugated secondary antibodies, or directly incubated with HRP-conjugated primary antibodies for chemiluminescence detection. The primary antibodies used for western blot analyses were anti-Flag HRP-conjugated (Sigma A8592), anti-β-Actin (Abcam 6276), anti-SETDB1 (Thermo Fisher Scientific MA5–15721), anti-SUV39H1 (Abcam ab12405), anti-SUV39H2 (Proteintech Group 11338–1-AP), anti-G9a (Sigma G6919), anti-GLP (Fisher Scientific PPB042200), anti-DNMT3b (Active Motif 39900), anti-DNMT3L (Sigma ABE2910), anti-TASOR (Sigma HPA006735), anti-HA Tag (Thermo Fisher Scientific 2–2.2.14), anti-H3 (Abcam ab1791), anti-UHRF1 (Santa Cruz sc-373750).

#### Total RNA Purification, reverse transcription (RT), and qPCR

Total RNA from mESCs or NPCs was isolated using TRIzol reagent (Invitrogen, #15596018), treated with TURBO DNase using TURBO DNA-free Kit (Invitrogen, #AM2238) and cleaned up further using RNeasy Mini kit purification (Qiagen) following the manufacturer’s protocol. Total RNA (1 μg) was reverse transcribed using random primers and Superscript III reverse transcriptase (Invitrogen) and quantified by qPCR on Applied Biosystems qPCR instrument in the presence of SYBR green with the primer sequences listed in Table S8 using the ΔCT method. *Gapdh* was used as an internal/loading control. Statistical analysis for RT-qPCR was performed on three biological replicates. Data in all figures are presented as mean values +/− S.D.

#### H3K9me3 ChIP-seq library preparation and high throughput sequencing

For ChIP-seq, reverse crosslinked DNA treated with RNase A and proteinase K was purified using phenol/chloroform/isoamyl alcohol and ethanol precipitated with 20 μg glycogen carrier. 10 ng of DNA was used to prepare libraries from two biological replicates per sample as described previously^110^. Libraries were pooled and sequenced on Illumina HiSeq platform. Single-end 150-bp raw reads were demultiplexed using the FASTX-Toolkit (version 0.0.13, http://hannonlab.cshl.edu/fastx_toolkit/). Reads were then aligned to the reference mouse genome (GRCm38/mm10, downloaded on January 4, 2020 from https://hgdownload.soe.ucsc.edu/goldenPath/mm10/bigZips/)^111^ using Bowtie2 (version 2.3.4.3)^112^ in local alignment mode with default parameters. To analyze H3K9me3 signal on the reporter gene, mm10 genome assembly was edited to include 2762 nt of the reporter sequence at its integration site on chromosome 2 (Chr2: 13574347). Chromosomal coordinates in the genome annotation (downloaded on January 13, 2021 from http://hgdownload.soe.ucsc.edu/goldenPath/mm10/bigZips/genes/mm10.ncbiRefSeq.gtf.gz) were edited accordingly to incorporate the reporter.

For H3K9me3 analysis at the reporter gene, duplicate reads were removed using Picard (version 2.8.0) (http://broadinstitute.github.io/picard/). For data visualization, aligned reads were normalized to counts per million using deepTools bamCoverage (version 3.0.2)^113^ and coverage values in bigwig files were computed using 10 bp bins. Genome tracks views were generated in the IGV genome browser^114^. Sequencing data (fastq and bigwig files) are available at the Gene Expression Omnibus (GEO) repository under accession number GSE212153.

#### Analysis of differentially methylated H3K9me3 peaks

To analyze differences in H3K9 trimethylation between *Uhrf1* KO and wild-type cells, we first defined H3K9me3 peaks/regions using epic2 (version 0.0.51)^62^ for each library with default parameters without removing duplicate reads (-kd) and with wild-type input for background normalization. To minimize biases in peak calling due to varying sequencing depth across libraries, we subsampled aligned reads using SAMtools (version >1.10)^115^ to get ~61 million reads per library prior to peak calling. Differential read enrichment within H3K9me3 peaks was analyzed using DiffBind (version 3.4.3)^63,116^ and edgeR. Finally, HOMER annotatePeaks (version 4.11.1)^117^ with default parameters was used to annotate all H3K9me3 peaks.

#### Analysis of H3K9me3 alterations on repeat elements

To analyze differences in H3K9me3 on repetitive genomic regions, reads were aligned to the mouse genome using Bowtie2 (ChIP-seq) as described above. Detailed repeat element annotations for the mouse genome were obtained from http://www.repeatmasker.org/species/mm.html (Repeat Library 20140131, downloaded on December 30, 2020). Repeat elements classified as “Simple repeat” or “Low complexity” were excluded from the analysis leaving 3,758,109 annotations comprising 1,363 uniquely named repeat elements (e.g., L1MdTf_I, IAPEY_LTR, MMSAT4, etc.) belonging to 65 repeat families (e.g., LINE/L1, LTR/ERVK, Satellite, etc.).

To analyze changes in H3K9me3 and RNA expression on repeat elements, we compared normalized read counts in wild-type versus *Uhrf1* KO cells for H3K9me3 ChIP-seq. Some repeat elements share high sequence identity (e.g., within the LINE/L1 family, L1MdTf_I, II, and III repeat consensus sequences share nearly 100% sequence identity), which can prove challenging to unambiguously align short reads to individual repeat elements. Considering this limitation, we assigned one alignment per read and summed up the total number of reads per repeat family to estimate methylation enrichment or RNA expression. We then divided normalized read counts per repeat family in *Uhrf1* KO by wild-type to get fold differences in expression.

Profile plot for GSAT_MM (major satellite sequence) was prepared by first aligning reads to 471 nt long GSAT_MM consensus sequence (DFAM reference id: DF0003028) using Bowtie2 in both wild-type and *Uhrf1* KO cells. The alignments were normalized by the total number of reads mapped to the genome (RPM) and converted to bigwig file format using deepTools bamCoverage as described above with a binsize of 1 (-binSize 1) and smoothing the coverage +/− 5 nt of each bin (--smoothLength 5). The profile plots show these alignments/bigwig files as visualized in IGV.

#### DNA CpG methylation analysis

Genomic DNA was purified from mESCs and NPCs and 1.5 μg of DNA was bisulfite converted using EpiTect Bisulfite Kit (Qiagen) according to manufacturer’s instructions. PCR products were amplified with EpiTect Methylation-specific PCR (MSP) kit (Qiagen) using the primers shown in Table S8, and then subcloned using TOPO Cloning (ThermoFisher) for sequencing. CpG methylation was analyzed with the BISMA tool (http://services.ibc.uni-stuttgart.de/BDPC/BISMA/) using the default parameters.

#### AlphaFold-Multimer structural predictions

Structural predictions in this study were performed as described earlier^98,101^. In brief, template-free mode of AlphaFold2-Multimer v3 was used to perform pairwise structural prediction with recycling number 5 using localColabFold at Harvard Medical School local computational cluster O2. Amino acid sequences used for structural predictions were obtained from UniProtKB database. Five possible structural models were provided by each prediction. Per-residue confidence score (pLDDT), predicted template modeling score (pTM) and interface predicted template modeling score (ipTM) of all five models were collected. Evaluation of the predicted structures were carried out first by plotting the first rank ipTM score heatmap and average ipTM score to visualize the confidence of the predicted protein-protein interactions. High confident predicted structures were then analyzed in UCSF Chimera X-1.6.1 to identify the predicted interaction interfaces. All pairwise predicted structures and customized structural predictions are included in Table S7. Predicted structures are available at ModelArchive through the accession ID: ma-dm-mmk9^118^.

#### GST pulldown assays

For bacteria protein expression, cDNA of the indicated protein domains was integrated to pGEX-6P-1 (GE Healthcare, 28-9546-48) with Gibson Assembly using the primers in Table S8. Proteins for GST pulldown assays were expressed in BL21 Codon Plus Escherichia coli with 200 μM isopropyl-β-D-thiogalactopyranoside (IPTG) induction at 18 °C for 12 hours. Bacteria were then collected and incubated in cold lysis buffer (50 mM Tris-HCl, pH 7.5, 400 mM NaCl, 10 mM KCl, 10 mM MgCl2, 0.2% Triton-X-100, 10% Glycerol, 1 mM DTT, protease inhibitor cocktail) for 2 hours at 4 °C. Cell extract with G9a protein was sonicated (Branson sonicator) for 18 seconds with 25% amplitude at 4 °C. After centrifuged at 20,000g for 10 min,the supernatant was incubated with 0.5 ml Glutathione Sepharose 4B resin (GE Healthcare, 17075605) for 2 h at 4 °C. The resin was then washed 6 times with the lysis buffer and eluted with reduced. To remove the GST tag, bead-coupled proteins were incubated with 3C-Protease in reaction buffer (50 mM Hepes, pH 7.9, 150 mM NaCl, 1 mM EDTA, 0.01% Triton-X-100, 10% Glycerol, 1 mM DTT, protease inhibitor cocktail) overnight at 4 °C. The GST-tagged 3C-Protease was removed using Glutathione Sepharose 4B resin. For GST pulldown assays, 10 μl 50% slurry of Glutathione Sepharose 4B was used for each sample. GST or GST-tagged proteins were incubated with untagged proteins in the binding buffer (50 mM Hepes, pH 7.9, 150 mM NaCl, 1 mM EDTA, 0.01% Triton-X-100, 10% Glycerol, 1 mM DTT, protease inhibitor cocktail) for 2 hours at 4 °C. Beads were washed 4 times with the binding buffer, resuspended in SDS protein buffer, and boiled for 5 min. Input and bound proteins were run on 4–20% gradient SDS–PAGE gel and analyzed by Coomassie blue staining.

#### Co-Immmunoprecipitation (Co-IP) assay

To prepare chromatin-enriched fractions, ~2 × 10^7^ mESCs (either untransduced or expressing HA-tagged DNMT3L WT or DNMT3L 3A mutant) were washed twice with ice-cold 1x PBS, gently scraped into 10 mL of ice-cold PBS, and pelleted by centrifugation at 1000 *g* for 5 min at 4°C. Nuclei were prepared by suspension of cell pellets in a hypotonic buffer (20 mM HEPES pH 7.9, 10 mM KCl, 1mM MgCl_2_, 0.1% Triton X-100, 20% Glycerol, 0.5mM DTT, 0.5mM PMSF, and protease inhibitors), and incubated on ice for 15 min to allow cell swelling. Nuclei were released by Dounce homogenization and pelleted by centrifugation at 1000 *g* for 5 min at 4°C. Nuclei were resuspended in nuclei lysis buffer (20 mM HEPES pH 7.9, 10 mM KCl, 1 mM MgCl_2_, 300 mM NaCl 0.1% Triton X-100, 20% Glycerol, 0.5 mM DTT, 0.5 mM PMSF, and protease inhibitors). Chromatin was sheared by sonication (55% amplitude, 2 pulses of 4 sec each, with samples kept on ice for 4 min between pulses) with a VibraCell sonicator (72434). Samples were then rotated (30 min, 4°C, 30 rpm) to solubilize chromatin. Lysates were diluted with nuclei lysis buffer (without NaCl) to a final concentration of 200 mM NaCl and clarified by centrifugation for 20mins at 15,000 *g* at 4°C. The soluble supernatant was incubated with anti-G9a antibody (Sigma G6919) and Protein A Dynabeads (Thermo fisher) for 3 hours at 4°C with rotation (15rpm). Beads were rinsed with ice-cold IP buffer (20 mM HEPES pH 7.9, 10 mM KCl, 1 mM MgCl_2_, 200 mM NaCl 0.1% Triton X-100, 20% Glycerol, 0.5 mM DTT, 0.5 mM PMSF, and protease inhibitors) three times. Samples/beads were boiled in SDS loading buffer for 5–10min before loaded to SDS-PAGE gel. Western blot was performed as described earlier.

#### Chromatin binding assay

Approximately 1 × 10^7^ mESCs (either untransduced or expressing HA-tagged DNMT3L WT or DNMT3L 3A mutant) were washed twice with ice-cold 1x PBS, scraped gently, and pelleted by centrifugation at 1000 *g* for 5 min at 4°C. Nuclei were prepared by suspension of cell pellets in a hypotonic buffer (20 mM HEPES pH 7.9, 10 mM KCl, 1mM MgCl2, 0.1% Triton X-100, 20% Glycerol, 0.5mM DTT, 0.5mM PMSF, and protease inhibitors), and incubated on ice for 15 min. After Dounce homogenization, nuclei were pelleted by centrifugation at 1000 *g* for 5 min at 4°C and resuspended in nuclei lysis buffer (as hypotonic buffer but with 300 mM NaCl). Chromatin was sheared by sonication (55% amplitude, 2 × 4 sec pulses, 4 min ice interval) and rotated (30 min, 4°C, 30 rpm). An aliquot was saved as nuclear fraction. After centrifugation (15,000 × *g*, 20 min, 4°C), supernatant (soluble fraction) was separated from pellet (chromatin fraction). Fractions were analyzed with Western blotting.

#### Quantification and statistical analysis

All statistical analyses were performed using GraphPad Prism (v9) for RT-qPCR, ChIP-qPCR and validation experiments and MAGeCK (v0.5.9.4) for CRISPR-Cas9 pooled screens. For ChIP-qPCR, RT-qPCR and validation experiments, statistical significance was determined using unpaired Student’s t tests. Data are presented as mean ± standard deviation (S.D.) unless otherwise indicated. The number of biological replicates (typically n = 3) is noted in the corresponding figure legends. For CRISPR-Cas9 pooled screens, statistical analysis was performed using the MAGeCK algorithm (v0.5.9.4). Log2 fold changes and p-values were calculated using a negative binomial model, and gene-level significance was determined using the modified robust ranking aggregation (α-RRA) method with Benjamini-Hochberg false discovery rate (FDR) correction. Z-scores for individual sgRNAs were calculated as: Z = (x – m)/s, where x is the sgRNA’s log2 fold change, m is the mean LFC across all sgRNAs, and s is the standard deviation. Gene-level Z-scores were calculated by averaging the Z-scores of all sgRNAs targeting the same gene. No statistical methods were used to predetermine sample size. Further statistical details, including the test used and the meaning of n, are annotated in the figure legends and Results section.

## Supplementary Material

1 Table S1. Epichromo library annotation, Related to STAR Methods

2 Table S2. Guide RNAs, Related to STAR Methods

3 Table S3. Establishment phase results, Related to Figure 2

4 Table S4. Maintenance phase results, Related to Figure 3

5 Table S5. Diffbind H3K9me3 Peaks Annotation, Related to Figure 5

6 Table S6. Repeats_H3K9me3, Related to Figure 5

7 Table S7. AlphaFold predictions, Related to STAR Methods

8 Table S8. List of primers, Related to STAR Methods

9

## Figures and Tables

**Table:** Key resources table

REAGENT or RESOURCE	SOURCE	IDENTIFIER
Antibodies		
Mouse monoclonal anti-Flag HRP-conjugated	Sigma-Aldrich	Cat# A8592; RRID: AB_439702
Mouse monoclonal anti-β-Actin (clone AC-15)	Abcam	Cat# ab6276; RRID: AB_2223210
Rabbit polyclonal anti-H3K9me3	Abcam	Cat# ab8898; RRID: AB_306848
Rabbit polyclonal anti-H3K27me3	Millipore	Cat# 17622
Rabbit monoclonal anti-H3K4me3 (clone MC315)	Sigma-Aldrich	Cat# 04-745; RRID: AB_1163444
Mouse monoclonal anti-FLAG M2	Sigma-Aldrich	Cat# F1804; RRID: AB_262044
Rabbit polyclonal anti-SETDB1	Proteintech	Cat# 11231-1-AP; RRID: AB_2186069
Rabbit polyclonal anti-SUV39H1	Diagenode	Cat# C15410368
Rabbit monoclonal anti-G9a (clone C6H3)	Cell Signal Technology	Cat# 3306S
Rabbit monoclonal anti-SUZ12 (clone D39F6)	Cell Signaling	Cat# 3737S
Mouse monoclonal anti-SETDB1 (clone 5H6D4)	Thermo Fisher Scientific	Cat# MA5-15721
Mouse monoclonal anti-SUV39H1 (clone 44.1)	Abcam	Cat# ab12405
Rabbit polyclonal anti-SUV39H2	Proteintech Group	Cat# 11338-1-AP
Rabbit polyclonal anti-G9a	Sigma-Aldrich	Cat# G6919
Mouse monoclonal anti-GLP (clone B0422)	Fisher Scientific	Cat# PPB042200
Rabbit polyclonal anti-DNMT3b	Active Motif	Cat# 39900
Rabbit polyclonal anti-DNMT3L	Sigma-Aldrich	Cat# ABE2910
Rabbit polyclonal anti-TASOR	Sigma-Aldrich	Cat# HPA006735
Rabbit polyclonal anti-H3	Abcam	Cat# ab1791
Mouse monoclonal anti-HA	Thermo Fisher Scientific	Cat# 26483
Goat polyclonal anti-Sox2	Biotechne	Cat# AF2018
Mouse monoclonal anti-UHRF1 (clone H-8)	Santa Cruz	Cat# sc-373750
Mouse monoclonal anti-HA	Thermo Fisher Scientific	Cat# 2-2.2.14
Bacterial and virus strains		
Endura electrocompetent cells	Lucigen	Cat# 60242-2
DH5a chemocompetent E.coli cells	This study	N/A
Stbl3 chemocompetent E.coli cells	This study	N/A
BL21 Codon Plus E.coli cells	This study	N/A
Chemicals, peptides, and recombinant proteins		
Gelatin	Sigma-Aldrich	Cat# G1890
DMEM	Thermo Fisher Scientific	Cat# 10313021
Fetal Bovine Serum (FBS)	Thermo Fisher Scientific	Cat# 10437
NEAA	Thermo Fisher Scientific	Cat# 11140
L-Glutamine	Thermo Fisher Scientific	Cat# 25030081
Penicillin/streptomycin	Thermo Fisher Scientific	Cat# 15140
B-merchaptoethanol	Sigma-Aldrich	Cat# 63689
Leukemia Inhibitory Factor (LIF)	StemCell Technologies	Cat# 78056
StemPro^®^ Accutase^®^ Cell Dissociation Reagent	Thermo Fisher Scientific	Cat# 1110501
Neurobasal^™^ Medium	Thermo Fisher Scientific	Cat# 21103-049
DMEM-F12	Thermo Fisher Scientific	Cat# 11330057
B27 Supplement (50X), serum free	Thermo Fisher Scientific	Cat# 17504044
N2 supplement	Thermo Fisher Scientific	Cat# 17502-048
Human FGF basic 154AA	Peprotech	Cat# 100-18B
Human EGF	StemCell Technologies	Cat# 78006.1
GlutaMAX^™^ Supplement	Thermo Fisher Scientific	Cat# 35050061
Doxycycline	Sigma-Aldrich	Cat# D-9891
Retinoic Acid	Sigma-Aldrich	Cat# R-2625
anti-adherence rinsing solution	StemCell Technologies	Cat# 07010
CHIR-99021	MedChemExpress	Cat# HY-10182G
PD0325901	MedChemExpress	Cat# HY-10254
Lipofectamine 2000	Thermo Fisher Scientific	Cat# 11668027
Lipofectamine 3000	Thermo Fisher Scientific	Cat# L3000015
Lipofectamine RNAiMAX	Thermo Fisher Scientific	Cat# 13778150
Opti-MEM I	Thermo Fisher Scientific	Cat# 31985070
SpCas9 recombinant protein	This Study	N/A
GlycoBlue	Thermo Fisher Scientific	Cat# AM9516
PEG-it Virus precipitation solution	System Biosciences	Cat# LV810A-1
G418 sulfate	Thermo Fisher Scientific	Cat# 11811031
Puromycin	Thermo Fisher Scientific	Cat# A1113803
Carbenicillin disodium salt	Sigma-Aldrich	Cat# C1389
Tango (10x) buffer	Thermo Fisher Scientific	Cat# BY5
Esp3I (BsmBI)	Thermo Fisher Scientific	Cat# ER0451
T7 DNA ligase	NEB	Cat# M0318S
Ex Taq^®^ DNA Polymerase	Takara	Cat# RR001A
Paraformaldehyde, 16%	Ted pella	Cat# 18505
Donkey Serum (Blocking)	Sigma-Aldrich	Cat# D9663
PMSF Protease Inhibitor	Thermo Fisher Scientific	Cat# 36978
cOmplete^™^, EDTA-free Protease Inhibitor Cocktail	Sigma-Aldrich	Cat# COEDTAF-RO
protease inhibitor cocktail	Sigma-Aldrich	Cat# P8215
Dynabeads^™^ Protein A	Invitrogen	Cat# 10002D
Dynabeads^™^ Protein G	Invitrogen	Cat# 10004D
Pierce^™^ 16% Formaldehyde (w/v), Methanol-free	Thermo Fisher Scientific	Cat# 28908
Proteinase K, recombinant, PCR Grade	Sigma-Aldrich	Cat# RPROTKSOL-RO
Phenol:Chloroform:Isoamyl Alcohol 25:24:1 Saturated with 10 mM Tris, pH 8.0, 1 mM EDTA	Sigma-Aldrich	Cat# P2069
Glycogen	Sigma-Aldrich	Cat# 10901393001
T4 DNA Polymerase	NEB	Cat# M0203L
T4 Polynucleotide kinase	NEB	Cat# M0201L
DNA Polymerase I, Large (Klenow) Fragment	NEB	Cat# M0210S
Klenow Fragment (3′→5′ exo–)	NEB	Cat# M0212L
Quick Ligase	NEB	Cat# M2200L
4–20% Mini-PROTEAN^®^ TGX Stain-Free^™^ Protein Gels	Biorad	Cat# 14568096, 4561093
IPTG	AmericanBio	Cat# AB00841-00050
Glutathione Sepharose 4B purification resin	GE Healthcare	Cat# 17075605
Taq DNA polymerase	This study	N/A
3C protease	This study	N/A
DTT	Sigma-Aldrich	Cat# 10708984001
NP-40	Thermo Fisher Scientific	Cat# 28324
Critical commercial assays		
GeneArt^™^ Precision gRNA Synthesis Kit	Thermo Fisher Scientific	Cat# A29377
Neon Transfection System	Thermo Fisher Scientific	Cat# MPK1025
2x NEBNext PCR Master Mix	New England BioLabs	Cat# M0541S
NucleoSpin^®^ Extract II	Clontech	Cat# 740609.250
HiSpeed^®^ Plasmid Maxi Kit	Qiagen	Cat# 12663
Donkey anti-Sheep IgG (H+L) Cross-Adsorbed Secondary Antibody, Alexa Fluor^™^ 594	Thermo Fisher Scientific	Cat# A-11016
Donkey anti-Sheep IgG (H+L) Cross-Adsorbed Secondary Antibody, Alexa Fluor^™^ 647	Thermo Fisher Scientific	Cat# A-21448
EpiTect Bisulfite Kit	Qiagen	Cat# 59104
EpiTect Methylation-specific PCR (MSP) kit	Qiagen	Cat# 59305
Zero Blunt^®^ TOPO^®^ PCR Cloning Kit	Thermo Fisher Scientific	Cat# 450245
Protran Nitrocellulose Hybridization Transfer Membrane, Pore size: 0.45 μm	Thermo Fisher Scientific	Cat# 45-004-002
SuperSignal^™^ West Pico PLUS Chemiluminescent Substrate	Thermo Fisher Scientific	Cat# 34580
TRIzol reagent	Invitrogen	Cat# 15596018
TURBO DNA-free Kit	Invitrogen	Cat# AM2238
RNeasy Mini Kit	Qiagen	Cat# 74104
SuperScript^™^ III Reverse Transcriptase	Thermo Fisher Scientific	Cat# 18080044
millTUBE 1 mL AFA fiber	Covaris	Cat# 520130
FACS tubes	Corning^™^	Cat# 352235
MiSeq Reagent Nano Kit v2 (300-cycles)	illumina	Cat# MS-103-1001
Deposited data		
CRISPR-Cas9 screen and EpiChromo Library	This study	GEO Accession# GSE212153, Tables S1, S2, S3, S4
Genome wide datasets (H3K9me3 ChIP-seq)	This study	GEO Accession# GSE212153, Tables S5, S6
AlphaFold predicted structures	This study	ModelArchive Accession ID: ma-dm-mmk9, Table S7
Raw data	This study	Mendeley Data: (https://data.mendeley.com/datasets/btdkb5nnnw/1, DOI: 10.17632/btdkb5nnnw.1)
Experimental models: Cell lines		
E14TG2a mouse ESCs	ATCC	Cat# CRL-1821
293 FT cells	Gibco	Cat# R70007
8x-tetO-eGFP reporter line in mESCs	This study	N/A
TetR-3xFlag-KRAB mESCs	This study	N/A
TetR-3xFlag-mESCs	This study	N/A
KDM4c-3xHA OE mESCs	This study	N/A
Uhrf1 knockout mESCs	This study	N/A
DNMT3L-3xHA WT mESCs	This study	N/A
DNMT3L-3xHA 3A mESCs	This study	N/A
NPCs derived from mESCs	This study	N/A
Oligonucleotides		
sgRNAs for genome editing	This study	TableS8
Cloning primers	This study	TableS8
qPCR primers	This study	TableS8
siRNAs sequences	This study	TableS8
Recombinant DNA		
lentiCRISPRv2	Addgene	Cat# 52961
iDuet101a	Addgene	Cat# 17629
pLV-tTRKRAB-red	Addgene	Cat# 12250
pSMART-HCKAN	Lucigen	Cat# 65316
8x-tetO-eGFP reporter plasmid	This study	N/A
TetR-3xFlag-KRAB donor plasmid	This study	N/A
TetR-3xFlag- donor plasmid	This study	N/A
pMD2.G (lentivirus packaging)	Addgene	Cat# 12259
psPAX2 (lentivirus packaging)	Addgene	Cat# 12260
sgRNA library plasmids (EpiChromo)	This study	TableS2
lentiCRISPRv2 with sgRNAs for validations	This study	TableS2
Fuw-dCas9-Dnmt3a-P2A-tagBFP	Addgene	Cat# 2527
Fuw-KDM4c-3xHA-P2A-Puro	This study	N/A
Fuw-DNMT3L WT-3xHA-P2A-Puro	This study	N/A
Fuw-DNMT3L 3A-3xHA-P2A-Puro	This study	N/A
Software and algorithms		
MAGeCK (v0.5.9.4)	Li et al. (2014)	RRID:SCR_021452
g:Profiler (version e106_eg53_p16_65fcd97)	Raudvere, U. et al. (2019)	RRID:SCR_006809
FASTX-Toolkit (version 0.0.13)	Hannon Lab, CSHL	RRID:SCR_005534
Bowtie2 (v2.3.4.3)	Langmead & Salzberg (2012)	RRID:SCR_016368
Picard (version 2.8.0)	Broad Institute	RRID:SCR_006525
DeepTools bamCoverage (version 3.0.2)	Ramirez, F., et al. (2014)	RRID:SCR_016366
SAMtools (version >1.10)	Li, H. et al. (2009)	RRID:SCR_002105
Epic2 (version 0.0.51)	Stovner, E. B. & Saetrom, P. (2019)	RRID:SCR_021150
DiffBind (v3.4.3)	Stark & Brown (2011)	RRID:SCR_012918
edgeR	Robinson et al. (2010)	RRID:SCR_012802
HOMER (v4.11.1)	Heinz et al. (2010)	RRID:SCR_010881
IGV	Robinson, J. T. et al. (2011)	RRID:SCR_011793
BISMA tool	Jacobs University Bremen	RRID:SCR_000688
UCSF ChimeraX	Goddard et al. (2018)	RRID:SCR_015872
Cytoscape (v3.9.0)	Shannon et al. (2003)	RRID:SCR_003032
FlowJo (v10.7.2)	BD Biosciences	RRID:SCR_008520
GraphPad Prism (v9)	GraphPad Software	RRID:SCR_002798
Nikon NIS Elements imaging software (Version 4.51)	Nikon	RRID:SCR_014329
localColabFold	Harvard Medical School O2 computing cluster	N/A
Other		
QuantStudio^™^ 7 Flex Real-Time PCR System, 384-well, desktop	Applied Biosystems	Cat# 4485701
Covaris E220 Focused-Ultrasonicator	Covaris	Cat# 500239
BD FACSAria II	BD Biosciences	Cat# 642510
BD FACSCalibur	BD Biosciences	Cat# 342975
iQue Screener PLUS	IntelliCyt	Cat# 11811
Nikon Eclipse Ts2 Microscope	Nikon	N/A
24 well Aggrewell 400 plates	StemCell Technologies	Cat# 34425
Vibra-Cell^™^ sonicator	Sonics and Materials	Cat# 72434

## Highlights

- Transiently induced H3K9me3 heterochromatin is epigenetically inherited in mESCs
- Heterochromatin becomes more stable upon cell differentiation
- CRISPR screens reveal requirements for heterochromatin establishment and maintenance
- H3K9me3 is maintained like an imprint but requires DNA methylation reinforcement
