# Supplementary material for: Requirements for establishment and epigenetic stability of mammalian heterochromatin
Source: Mol Cell. Author manuscript; Available in PMC 2025 Sep 29. (PMC12478525; doi:10.1016/j.molcel.2025.08.025)
Supplement: 9 [file NIHMS2110126-supplement-9.pdf]

Figure S1

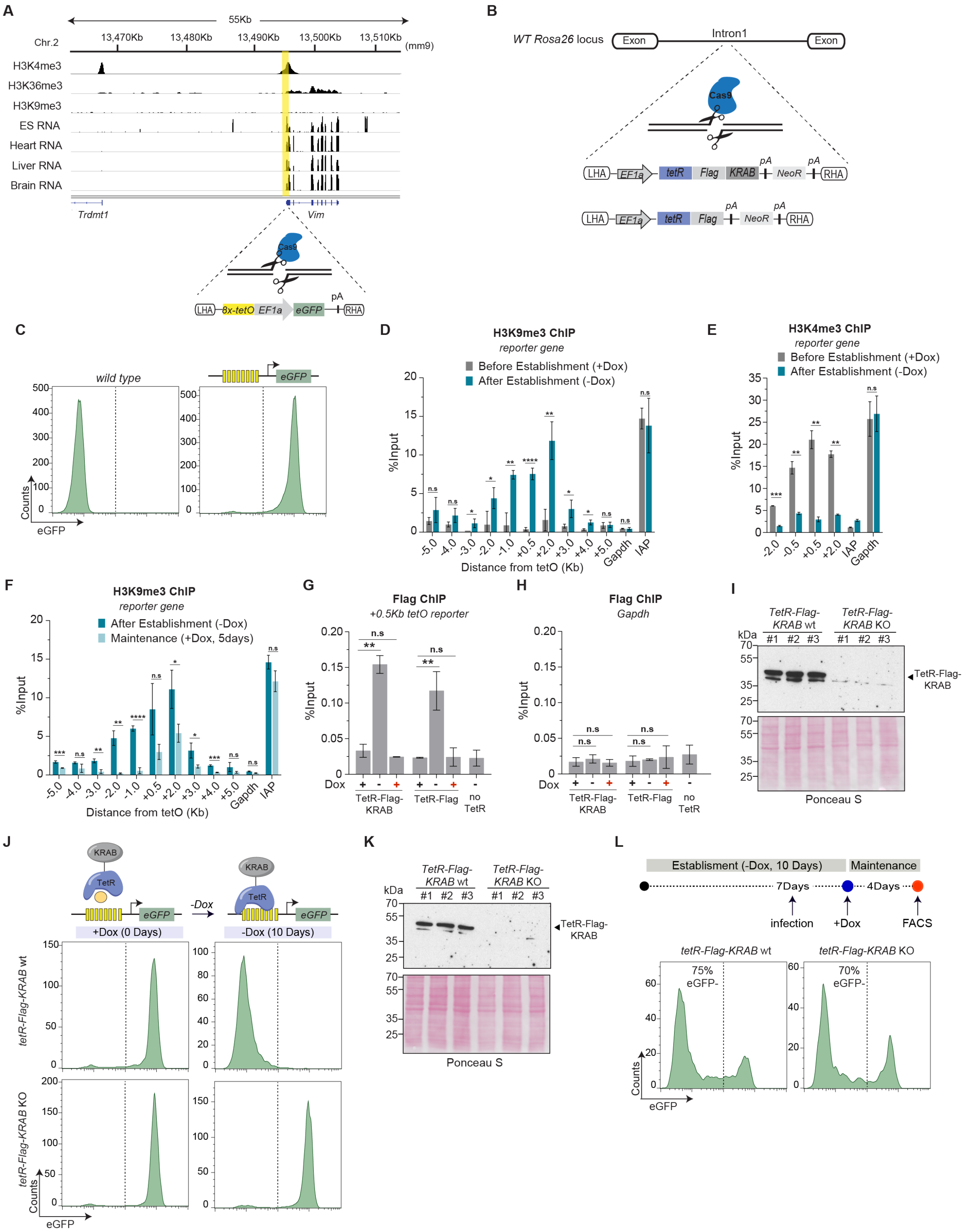
A
55Kb
Chr.2
13,470Kb
13,480Kb
13,490Kb
13,500Kb
13,510Kb
(mm9)
H3K4me3
H3K36me3
H3K9me3
ES RNA
Heart RNA
Liver RNA
Brain RNA
Trdmt1
Vim
Cas9
LHA
8x-tetO
EF1a
eGFP
pA
RHA
B
WT Rosa26 locus
Exon
Intron1
Exon
Cas9
LHA
EF1a
tetR
Flag
KRAB
pA
NeoR
pA
RHA
C
wild type
eGFP
Counts
eGFP
D
H3K9me3 ChIP
reporter gene
Before Establishment (+Dox)
After Establishment (-Dox)
%Input
Distance from tetO (Kb)
E
H3K4me3 ChIP
reporter gene
Before Establishment (+Dox)
After Establishment (-Dox)
%Input
F
H3K9me3 ChIP
reporter gene
After Establishment (-Dox)
Maintenance (+Dox, 5days)
%Input
Distance from tetO (Kb)
G
Flag ChIP
+0.5Kb tetO reporter
%Input
Dox
TetR-Flag-KRAB
TetR-Flag
no TetR
H
Flag ChIP
Gapdh
%Input
Dox
TetR-Flag-KRAB
TetR-Flag
no TetR
I
TetR-Flag-KRAB wt
TetR-Flag-KRAB KO
kDa
#1 #2 #3
#1 #2 #3
TetR-Flag-KRAB
Ponceau S
J
KRAB
TetR
eGFP
-Dox
+Dox (0 Days)
-Dox (10 Days)
tetR-Flag-KRAB wt
tetR-Flag-KRAB KO
Counts
eGFP
K
TetR-Flag-KRAB wt
TetR-Flag-KRAB KO
kDa
#1 #2 #3
#1 #2 #3
TetR-Flag-KRAB
Ponceau S
L
Establisment (-Dox, 10 Days)
Maintenance
7Days
4Days
infection
+Dox
FACS
tetR-Flag-KRAB wt
tetR-Flag-KRAB KO
75% eGFP-
70% eGFP-
Counts
eGFP

Figure S1. Establishment and maintenance of an inducible H3K9me3 domain and silencing at a reporter locus in mESCs. Related to Figure 1.

(A) Histone modifications and RNA expression levels in E14 mESCs and RNA levels in different mouse tissues at the genomic region where *8x-tetO-eGFP* reporter gene was inserted located on chromosome 2. Data from the ENCODE project are displayed and the genome coordinates are based on mm9 (MGSCv37) genome assembly. The *8x-tetO-eGFP* reporter gene was integrated via HDR-mediated CRISPR-Cas9 at the promoter of the vimentin gene (Vim) as depicted below the tracks. LHA and RHA, left and right homology arms; pA, signal. **(B)** Schematic diagram depicting the strategy and integration site of TetR-Flag-KRAB and TetR-Flag in the first intron at the *Rosa26* locus located on chromosome 6 in mESCs. **(C)** Flow cytometry histograms show eGFP expression in wild-type and *8x-tetO-eGFP* mESCs in the absence of TetR fusion proteins. **(D)** ChIP-qPCR analysis for H3K9me3 at the *8x-tetO-eGFP* reporter locus and surrounding regions in mESCs expressing TetR-Flag-KRAB cultured in the presence (Before Establishment, +Dox) or absence (After Establishment, -Dox) of doxycycline. *Gapdh* and *IAP*, are used as controls for euchromatin and heterochromatin H3K9me3 levels respectively. Values are shown as percentage (%) of input. Error bars, standard deviation (SD); n = 3 replicates. **(E)** Same as in **D** for H3K4me3 ChIP. **(F)** Same as in **D** for H3K9me3 levels in mESCs cultured in the absence (After Establishment, -Dox) or presence for 5 days of doxycycline (Maintenance, +Dox). **(G)** ChIP-qPCR analysis for TetR-Flag-KRAB and TetR-Flag binding at the *8x-tetO-eGFP* reporter locus before establishment (+Dox), after establishment (-Dox), and during the maintenance phase of silencing after adding back doxycycline to the medium for 24 hours (+Dox, in red). *8x-tetO-eGFP* reporter mESC line without TetR-Flag-KRAB or TetR-Flag was used as a negative control. Values are shown as percentage (%) of input. Error bars, standard deviation (SD); n = 3 biological replicates. **(H)** Same as in **G** at the *Gapdh* promoter. **(I)** Western blot (top) showing protein levels of TetR-Flag-KRAB before (*TetR-Flag-KRAB* wt) and after its deletion (*TetR-Flag-KRAB* KO) in mESCs cultured in the presence of doxycycline before establishment of silencing. Ponceau S staining used as a loading control (bottom). Molecular weights in kilodalton are shown on the left. **(J)** Flow cytometry histograms showing eGFP expression in *tetR-Flag-KRAB* wt and *tetR-Flag-KRAB* KO mESCs cultured with and without doxycycline (-Dox). **(K)** Same as in **I** but TetR-Flag-KRAB was deleted after establishment of silencing. **(L)** Same as in **J** showing eGFP

expression in mESCs with the indicated genotypes. Top, diagram of experimental strategy. Cells were cultured for seven days without doxycycline (-Dox), followed with infection with two lentiviral vectors carrying two sgRNAs targeting the TetR domain of TetR-Flag-KRAB, three days later doxycycline (+Dox) was added back for four days. Bottom, Flow cytometry histograms showing similar maintenance of silencing in in *tetR-Flag-KRAB* wt and *tetR-Flag-KRAB* KO mESCs. *P*-values in panels **D**-**H** calculated with two-tailed unpaired Student's t-tests are indicated with asterisks. n.s, not significant ($P > 0.05$), *$P \leq 0.05$, **$P < 0.01$, ***$P < 0.001$, ****$P < 0.0001$.

Figure S2

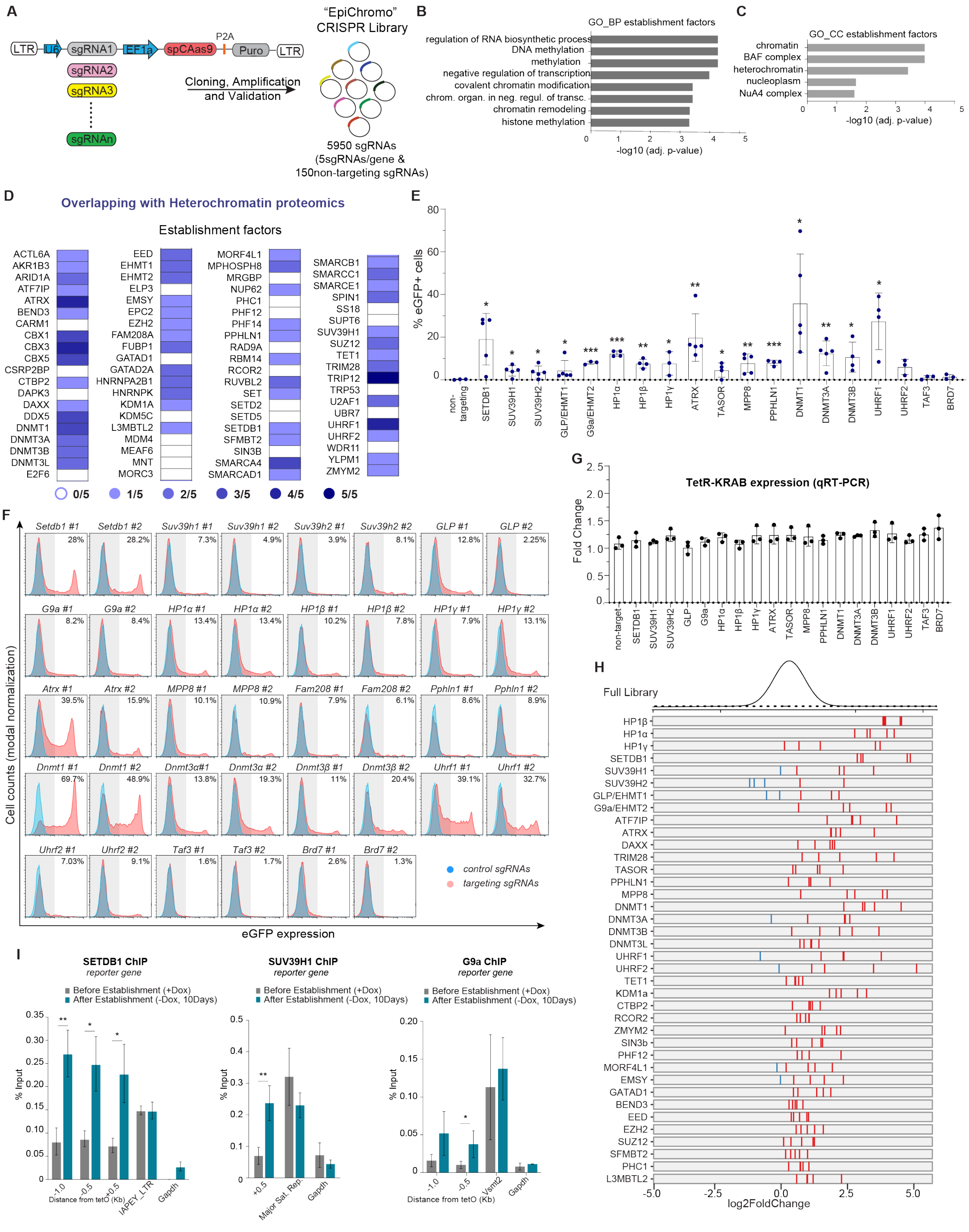

A
"EpiChromo" CRISPR Library
LTR U6 sgRNA1 EF1a spCAas9 P2A Puro LTR
sgRNA2
sgRNA3
sgRNAn
Cloning, Amplification and Validation
5950 sgRNAs (5sgRNAs/gene & 150non-targeting sgRNAs)
B
GO_BP establishment factors
regulation of RNA biosynthetic process
DNA methylation
methylation
negative regulation of transcription
covalent chromatin modification
chrom. organ. in neg. regul. of transc.
chromatin remodeling
histone methylation
-log10 (adj. p-value)
C
GO_CC establishment factors
chromatin
BAF complex
heterochromatin
nucleoplasm
NuA4 complex
-log10 (adj. p-value)
D
Overlapping with Heterochromatin proteomics
Establishment factors
0/5
1/5
2/5
3/5
4/5
5/5
E
% eGFP+ cells
F
Cell counts (modal normalization)
eGFP expression
control sgRNAs
targeting sgRNAs
G
TetR-KRAB expression (qRT-PCR)
Fold Change
H
Full Library
log2FoldChange
I
SETDB1 ChIP
reporter gene
SUV39H1 ChIP
reporter gene
G9a ChIP
reporter gene
Before Establishment (+Dox)
After Establishment (-Dox, 10Days)
% Input
Distance from tetO (Kb)
IAPEY_LTR
Gapdh
Major Sat. Rep.
Vsmt2

Figure S2. Supplemental of establishment screen data. Related to Figure 2.

(A) Schematic diagram showing the targeting vector and strategy followed to generate the EpiChromo library used to perform the pooled screens. **(B)** Gene ontology (GO_BP) analysis for the biological processes associated with the 79 establishment factors. **(C)** Gene ontology (GO_CC) analysis for the cellular components associated with the 79 establishment factors. Of note, the NuA4 complex shares components with HDAC complexes likely explaining its appearance on the list of candidate factors. **(D)** Heatmaps showing overlap of establishment factors with heterochromatin proteomics data. Color intensity scales with the number of datasets identifying each protein, with darker hues indicating higher consensus across studies. Out of 79 establishment factors, 55 (70%) overlapped with at least one proteomics dataset. This overlap represents a statistically significant enrichment relative to the overall library background (P-value $\approx 7.0 \times 10^{-7}$, hypergeometric test). Source data for this figure are provided in Table S3. **(E)** Validation of candidate genes from the screen associated with H3K9 methylation and DNA methylation was performed nine days after viral-mediated sgRNA transduction. The indicated genes were targeted by three to five sgRNAs individually and eGFP expression was measured by FACS. Percentage (%) of $eGFP^+$ cells are presented. Mean values are shown, error bars are SD; n=3-5 sgRNAs each gene. P-values calculated with unpaired Student's t-tests compared to control sgRNAs are indicated with asterisks. n.s., not significant ($P > 0.05$), *$P \leq 0.05$, **$P < 0.01$, ***$P < 0.001$. **(F)** Flow cytometry histograms (eGFP on the x-axis) for the top two sgRNAs per targeted gene, as shown in panel **E**. The percentage of single cells with eGFP expression (eGFP+) is indicated for each sgRNA. **(G)** qRT-PCR experiment measuring the expression levels of TetR-KRAB fusion protein in cells transduced with sgRNAs targeting the indicated genes as in panel **E**. **(H)** Performance in the pooled screen of sgRNAs targeting the 38 genes depicted in **Figure 2E**. Log_2 fold change in $eGFP^+$ sorted versus unsorted cells is shown for the full library of sgRNAs (top) and for the 5 sgRNAs targeting each gene. sgRNAs with positive or negative log_2 fold change are shown in red or blue respectively. **(I)** ChIP-qPCR analysis for SETDB1, SUV39H1 and G9a binding at the *8x-tetO-eGFP* reporter locus in mESCs before establishment, and after establishment of silencing. Values are shown as percentage (%) of input. *Gapdh* promoter is used as a negative control region. IAPEY_LTR, Major Satellite Repeats and *Vsmt2* gene are used as positive control regions for SETDB1, SUV39H1 and G9a binding respectively. Error bars, standard deviation (SD); n = 3 biological replicates.

Figure S3

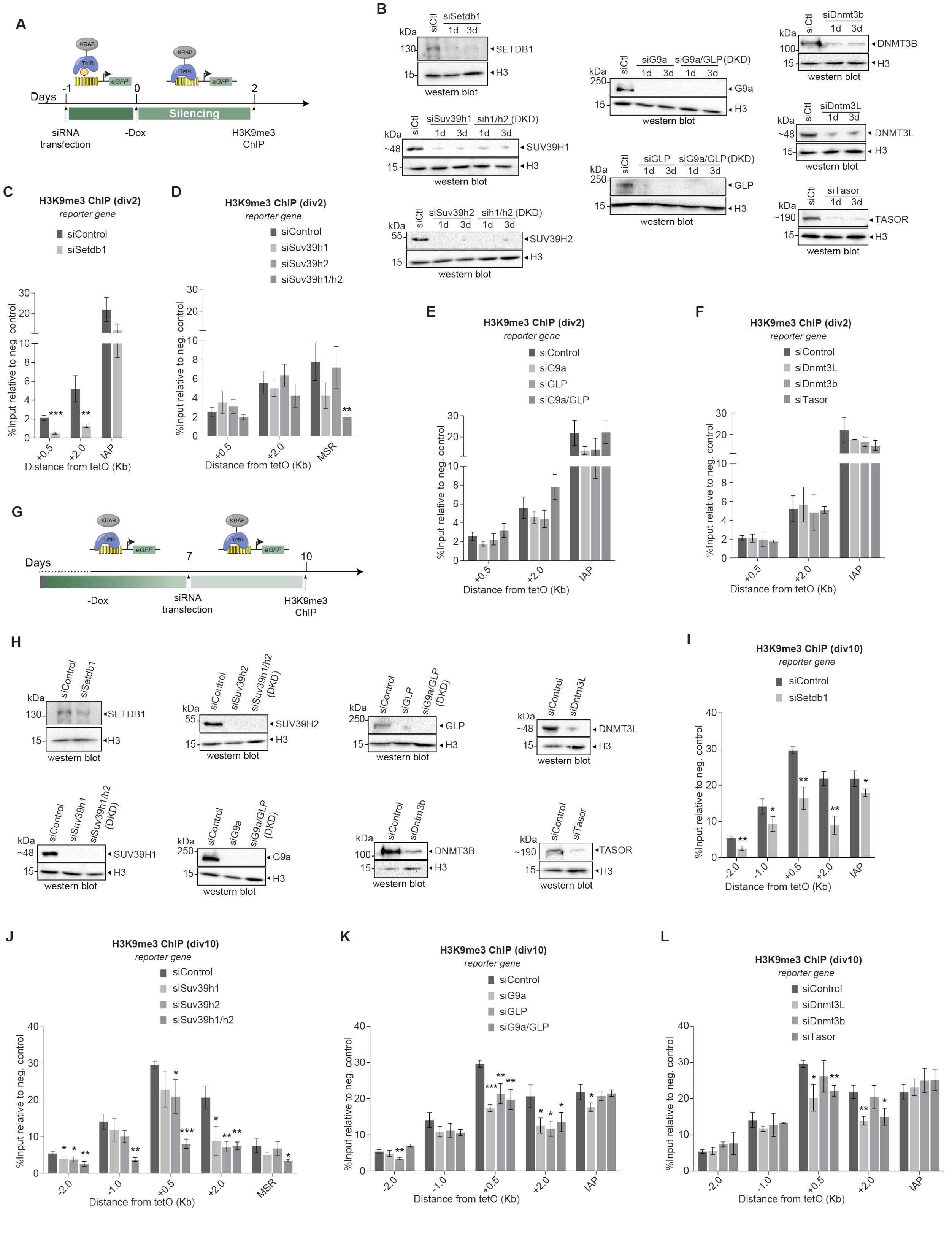

Figure S3. Effects on H3K9me3 levels following targeted knockdowns. Related to Figure 2. **(A)** Outline of the assay to test H3K9me3 levels at the reporter locus in mESCs upon siRNA-mediated knockdown of several factors during early days of establishment (div2). **(B)** Western blots showing (top) protein levels of the indicated factors in mESCs transfected with control siRNA (siCtl) or siRNAs targeting specific factors, analyzed at one and three days post-transfection, and H3 western blots used as a loading control (bottom). Experimental conditions follow the outline in **Figure S3A**. (DKD) indicate double knockdowns. Molecular weights in kilodalton are indicated on the left. **(C-F)** ChIP-qPCR analysis for H3K9me3 at the *8x-tetO-eGFP* reporter locus and IAP elements or the Major Satellite Repeats (MSR) in mESCs transfected with control or the indicated siRNAs cultured in the absence of doxycycline for two days from the onset of establishment (div2). Values are shown relative to *Gapdh* used as a negative control region. Error bars, standard deviation (SD); n = 3 replicates. **(G)** Outline of the assay to test H3K9me3 levels at the reporter locus in mESCs upon siRNA-mediated knockdown of several factors later during establishment (div10). **(H)** Western blots showing (top) protein levels of the indicated factors in mESCs transfected with control siRNA (siControl) or siRNAs targeting specific factors, analyzed at three days post-transfection, and H3 western blots used as a loading control (bottom). Experimental conditions follow the outline in **Figure S3G**. (DKD) indicate double knockdowns. Molecular weights in kilodalton are indicated on the left. **(I-L)** ChIP-qPCR analysis for H3K9me3 at the *8x-tetO-eGFP* reporter locus and IAP elements or MSR in mESCs transfected with control or the indicated siRNAs following the outline in panel **G**. Values are shown relative to *Gapdh* used as a negative control region. Error bars, standard deviation (SD); n = 3 replicates. *P*-values calculated by two-tailed unpaired Student's t-tests are indicated with asterisks. n.s., not significant ($P > 0.05$), *$P \leq 0.05$, **$P < 0.01$, ***$P < 0.001$.

Figure S4

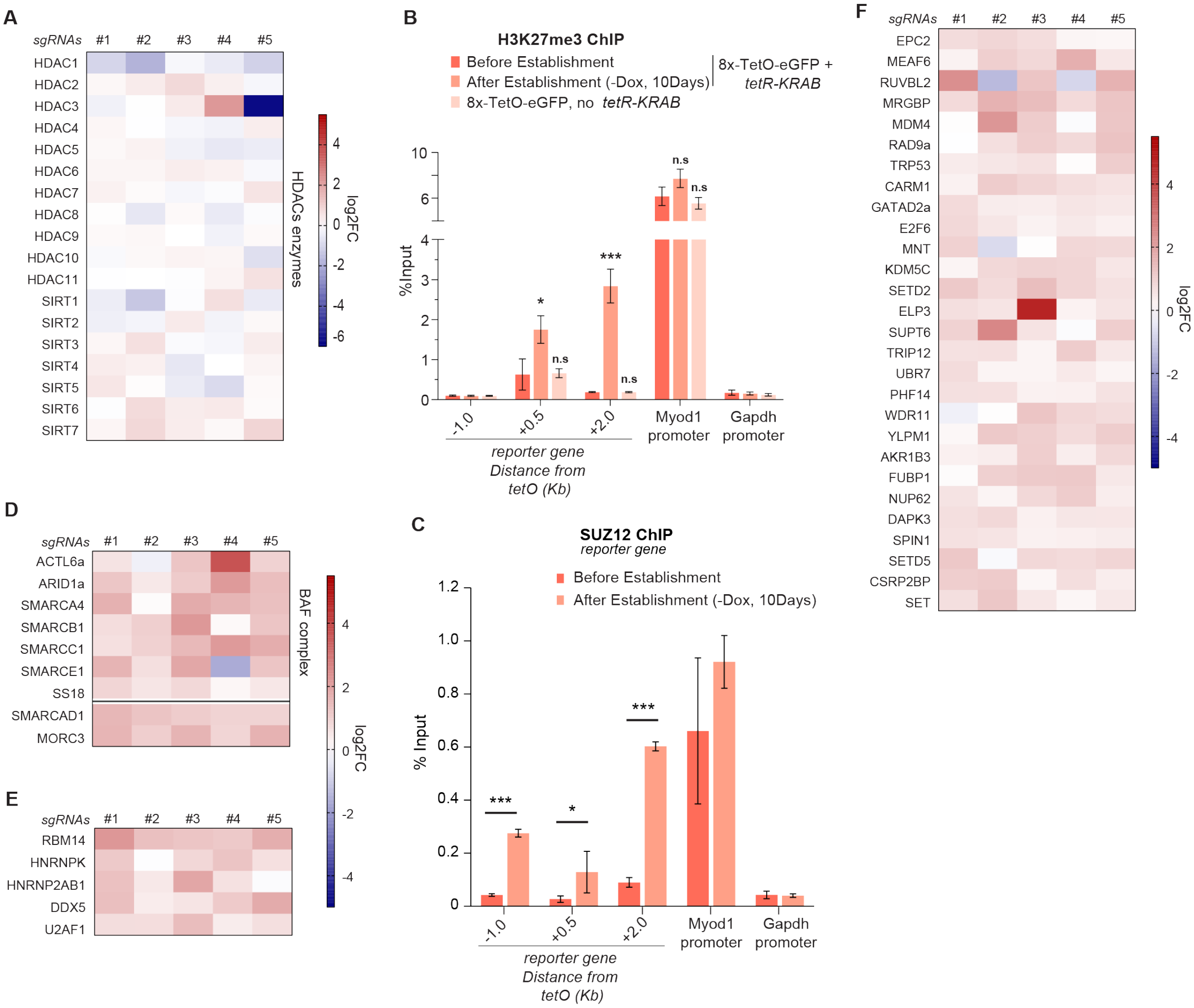

Figure S4. Supplemental of establishment screen data. Related to Figure 2.

(A) Heatmap depicting HDAC enzymes (left) affecting establishment of silencing. Log_2 fold change of each one of the 5 sgRNAs targeting each gene in $eGFP^+$ sorted versus unsorted cells is shown (n = two independent replicates). **(B)** ChIP-qPCR analysis for H3K27me3 at the *8x-tetO-eGFP* reporter locus in mESCs before establishment, after establishment of silencing and in *8x-tetO-eGFP* mESCs without TetR-Flag-KRAB. Values are shown as percentage (%) of input. *Myod1* and *Gapdh* promoters are used as H3K27me3 positive and negative control regions respectively. Error bars, standard deviation (SD); n = 3 biological replicates. *P*-values calculated by two-tailed unpaired Student's t-tests with (Before Establishment) condition are indicated with asterisks. n.s., not significant ($P > 0.05$), $*P \leq 0.05$, $**P < 0.01$, $***P < 0.001$. **(C)** ChIP-qPCR

analysis for SUZ12 binding at the *8x-tetO-eGFP* reporter locus in mESCs before establishment, and after establishment of silencing. Values are shown as percentage (%) of input. *Myod1* and *Gapdh* promoters are used as positive and negative control regions respectively. Error bars, standard deviation (SD); n = 3 biological replicates. **(D-F)** Same as in **A** for chromatin remodelers, RNA processing factors and cluster 8 genes respectively. *P*-values in panels A, C and D calculated with two-tailed unpaired Student's t-tests are indicated with asterisks. n.s, not significant ($P > 0.05$), *$P \leq 0.05$, **$P < 0.01$, ***$P < 0.001$.

Figure S5

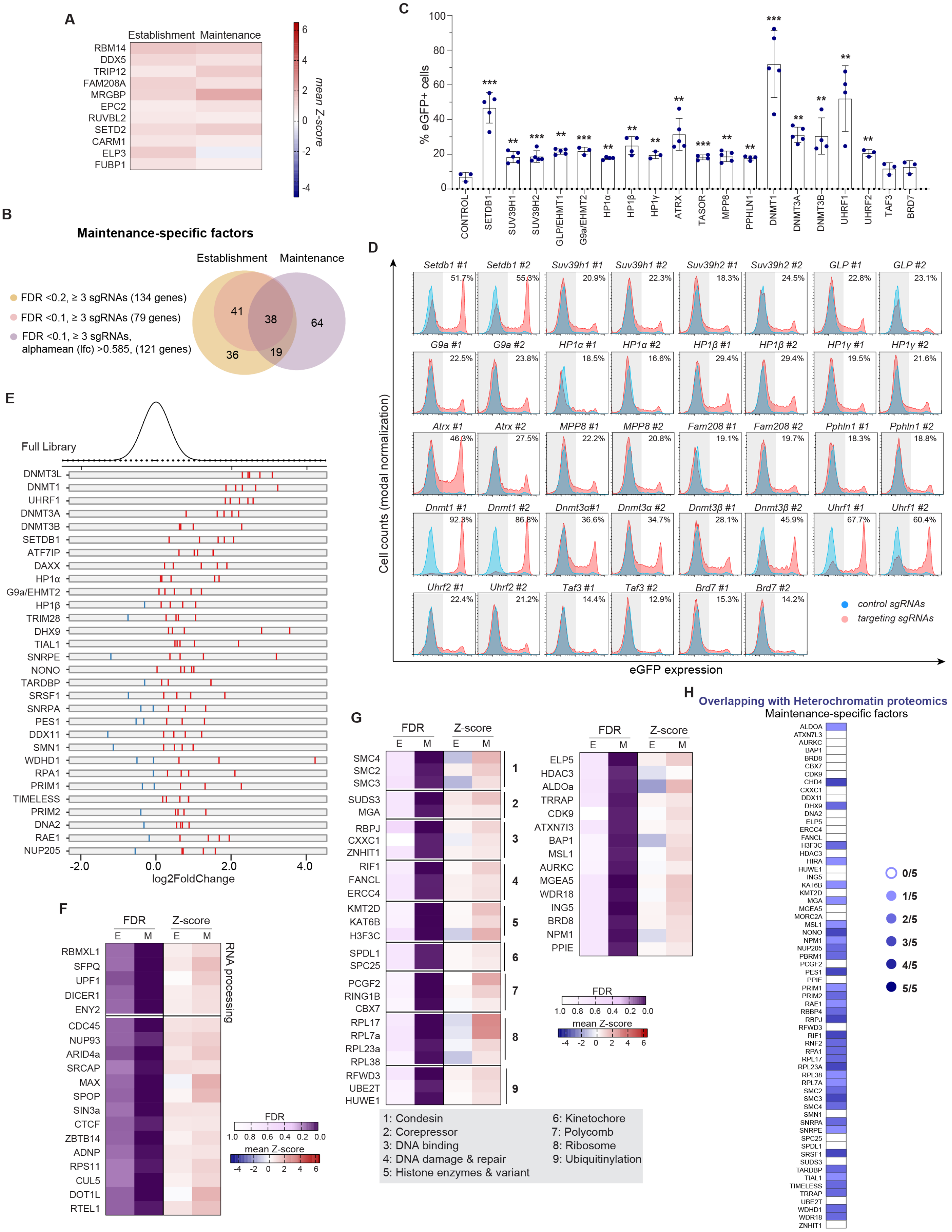
A
Establishment Maintenance
RBM14
DDX5
TRIP12
FAM208A
MRGBP
EPC2
RUVBL2
SETD2
CARM1
ELP3
FUBP1
mean Z-score
6
4
2
0
-2
-4
B
Maintenance-specific factors
Establishment Maintenance
FDR <0.2, ≥ 3 sgRNAs (134 genes)
FDR <0.1, ≥ 3 sgRNAs (79 genes)
FDR <0.1, ≥ 3 sgRNAs, alphamean (lfc) >0.585, (121 genes)
41
38
64
36
19
C
% eGFP+ cells
100
80
60
40
20
0
CONTROL
SETDB1
SUV39H1
SUV39H2
GLP/EHMT1
G9a/EHMT2
HP1α
HP1β
HP1γ
ATRX
TASOR
MPP8
PPHLN1
DNMT1
DNMT3A
DNMT3B
UHRF1
UHRF2
TAF3
BRD7
D
Cell counts (modal normalization)
eGFP expression
Setdb1 #1 51.7%
Setdb1 #2 55.3%
Suv39h1 #1 20.9%
Suv39h1 #2 22.3%
Suv39h2 #1 18.3%
Suv39h2 #2 24.5%
GLP #1 22.8%
GLP #2 23.1%
G9a #1 22.5%
G9a #2 23.8%
HP1α #1 18.5%
HP1α #2 16.6%
HP1β #1 29.4%
HP1β #2 29.4%
HP1γ #1 19.5%
HP1γ #2 21.6%
Atrx #1 46.3%
Atrx #2 27.5%
MPP8 #1 22.2%
MPP8 #2 20.8%
Fam208 #1 19.1%
Fam208 #2 19.7%
Pphln1 #1 18.3%
Pphln1 #2 18.8%
Dnmt1 #1 92.8%
Dnmt1 #2 86.8%
Dnmt3α#1 36.6%
Dnmt3α #2 34.7%
Dnmt3β #1 28.1%
Dnmt3β #2 45.9%
Uhrf1 #1 67.7%
Uhrf1 #2 60.4%
Uhrf2 #1 22.4%
Uhrf2 #2 21.2%
Taf3 #1 14.4%
Taf3 #2 12.9%
Brd7 #1 15.3%
Brd7 #2 14.2%
control sgRNAs
targeting sgRNAs
E
Full Library
DNMT3L
DNMT1
UHRF1
DNMT3A
DNMT3B
SETDB1
ATF7IP
DAXX
HP1α
G9a/EHMT2
HP1β
TRIM28
DHX9
TIAL1
SNRPE
NONO
TARDBP
SRSF1
SNRPA
PES1
DDX11
SMN1
WDHD1
RPA1
PRIM1
TIMELESS
PRIM2
DNA2
RAE1
NUP205
-2.0
0.0
2.0
4.0
log2FoldChange
F
FDR Z-score
E M E M
RBMXL1
SFPQ
UPF1
DICER1
ENY2
CDC45
NUP93
ARID4a
SRCAP
MAX
SPOP
SIN3a
CTCF
ZBTB14
ADNP
RPS11
CUL5
DOT1L
RTEL1
RNA processing
FDR
1.0 0.8 0.6 0.4 0.2 0.0
mean Z-score
-4 -2 0 2 4 6
G
FDR Z-score
E M E M
SMC4
SMC2
SMC3
SUDS3
MGA
RBPJ
CXXC1
ZNHIT1
RIF1
FANCL
ERCC4
KMT2D
KAT6B
H3F3C
SPDL1
SPC25
PCGF2
RING1B
CBX7
RPL17
RPL7a
RPL23a
RPL38
RFWD3
UBE2T
HUWE1
1
2
3
4
5
6
7
8
9
ELP5
HDAC3
ALDOa
TRRAP
CDK9
ATXN7I3
BAP1
MSL1
AURKC
MGEA5
WDR18
ING5
BRD8
NPM1
PPIE
FDR
1.0 0.8 0.6 0.4 0.2 0.0
mean Z-score
-4 -2 0 2 4 6
1: Condesin
2: Corepressor
3: DNA binding
4: DNA damage & repair
5: Histone enzymes & variant
6: Kinetochore
7: Polycomb
8: Ribosome
9: Ubiquitinylation
H
Overlapping with Heterochromatin proteomics
Maintenance-specific factors
ALDOA
ATXN7L3
AURKC
BAP1
BRD8
CBX7
CDK9
CHD4
CXXC1
DDX11
DHX9
DNA2
ELP5
ERCC4
FANCL
H3F3C
HDAC3
HIRA
HUWE1
ING5
KAT6B
KMT2D
MGA
MGEA5
MORC2A
MSL1
NONO
NPM1
NUP205
PBRM1
PCGF2
PES1
PPIE
PRIM1
PRIM2
RAE1
RBBP4
RBPJ
RFWD3
RIF1
RNF2
RPA1
RPL17
RPL23A
RPL38
RPL7A
SMC2
SMC3
SMC4
SMN1
SNRPA
SNRPE
SPC25
SPDL1
SRSF1
SUDS3
TARDBP
TIAL1
TIMELESS
TRRAP
UBE2T
WDHD1
WDR18
ZNHIT1
0/5
1/5
2/5
3/5
4/5
5/5

Figure S5. Analysis of maintenance screen results. Related to Figure 3.

(A) Heatmap of 11 out of the 38 gene hits (left) that affect both establishment and maintenance of silencing and are not presented in **Figure 3D**. Mean values of the Z-scores of all 5 sgRNAs targeting each gene in $eGFP^+$ sorted versus unsorted cells are shown in each condition (n = two independent replicates). **(B)** Venn diagram showing the number of genes affecting the establishment, maintenance, or both phases of silencing of the reporter locus. Alphamean, means of log2 fold change of the effective sgRNAs. **(C)** Validation of selected candidate genes required for maintenance and associated with H3K9 and DNA methylation was done three days after the addition of DOX and twelve days after viral-mediated sgRNA transduction. The indicated genes were targeted by three to five sgRNAs individually and eGFP expression was assessed by FACS. Percentage (%) of $eGFP^+$ cells is indicated (y-axis). Mean values are shown, error bars are SD; n=3-5 sgRNAs for each gene. *P*-values calculated with unpaired Student's t-tests compared to control sgRNAs are indicated with asterisks. n.s., not significant ($P > 0.05$), *$P \leq 0.05$, **$P < 0.01$, ***$P < 0.001$. **(D)** Flow cytometry histograms (eGFP on the x-axis) for the top two sgRNAs per targeted gene, as shown in panel **B**. The percentage of single cells with eGFP expression (eGFP+) is indicated for each sgRNA. **(E)** Performance in the pooled screen of sgRNAs targeting the gene hits shown (left). Log_2 fold change in $eGFP^+$ sorted versus unsorted cells is shown for the full library of sgRNAs (top) and for the 5 sgRNAs targeting each gene. sgRNAs with positive or negative log_2 fold change are shown in red or blue respectively. **(F)** Heatmap of the 19 gene hits found to affect both maintenance (FDR < 0.1) and establishment but at a lower cutoff (FDR < 0.2). Gene-level FDR values and mean values of the Z-scores of all 5 sgRNAs targeting each gene in $eGFP^+$ sorted versus unsorted cells during establishment (E) or maintenance (M) are shown. (n = two independent replicates). **(G)** Same as in **F** showing gene hits (left) belonging to the indicated functional categories (right) that are maintenance-specific regulators of silencing. **(H)** Heatmaps showing overlap of maintenance-specific factors with heterochromatin proteomics data. Color intensity scales with the number of datasets identifying each protein, with darker hues indicating higher consensus across studies. This overlap represents a statistically significant enrichment relative to the overall library background (*P*-value ≈ 0.0102, hypergeometric test). Source data for this figure are provided in Table S4.

Figure S6

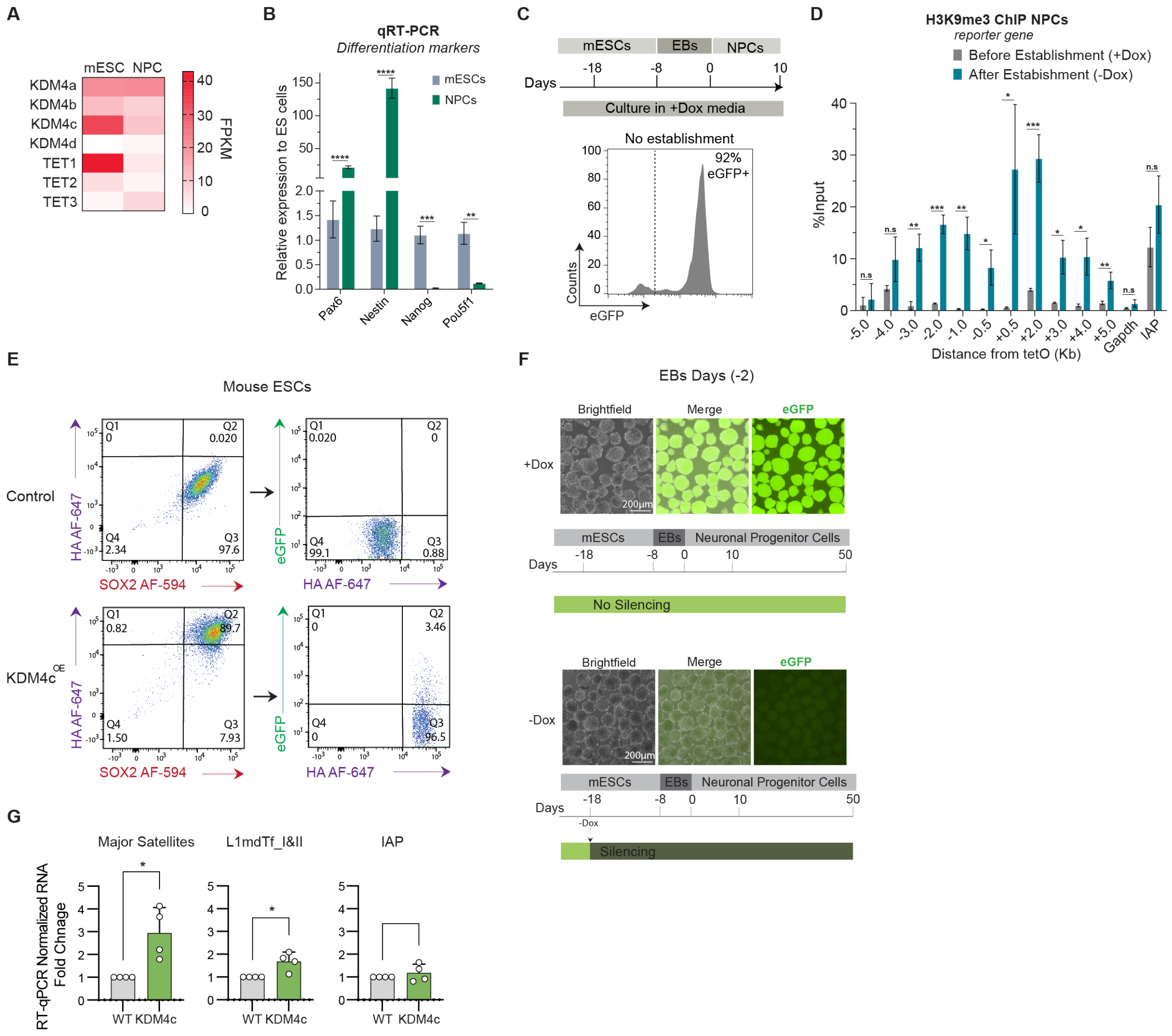

Figure S6. Epigenetic maintenance of silencing is stabilized upon differentiation. Related to Figure 4.

(A) Heatmap showing expression levels of KDM4s and TETs in mESCs and NPCs quantified by RNA-seq in Terranova et al. (2015)[56]. Values are displayed as fragments per kilobase of transcript per million fragments mapped (FPKM). **(B)** qRT-PCR analysis showing increased expression levels of neural progenitor cell markers (*nestin, pax6*) and decreased levels of embryonic stem cell related genes (*nanog, pou5f1*) after differentiation of *8x-tetO-eGFP/TetR-Flag-KRAB* reporter mESCs to NPCs. Mean values are shown. Error bars, standard deviation (SD); n = 3 biological replicates. *P*-values calculated by two-tailed unpaired Student's t-tests are

indicated with asterisks. n.s., not significant (P > 0.05), *P ≤ 0.05, **P < 0.01, ***P < 0.001. **(C)** Flow cytometry histogram shows eGFP expression in neural progenitor cells that were differentiated from mESCs in the continuous presence of doxycycline (+Dox). Percentage (%) indicates fraction of $eGFP^+$ cells. **(D)** ChIP-qPCR analysis for H3K9me3 at the *8x-tetO-eGFP* reporter locus and surrounding regions in neural progenitor cells (NPCs) 10 days after plating (EB dissociation) cultured in the presence (Before Establishment, +Dox) or absence (After Establishment, -Dox) of doxycycline. *Gapdh* and *IAP*, are used as controls for euchromatin and heterochromatin H3K9me3 levels respectively. Values are shown as percentage (%) of input. Error bars, standard deviation (SD); n = 3 replicates. *P*-values calculated by two-tailed unpaired Student's t-tests are indicated with asterisks. n.s., not significant (*P* > 0.05), **P* ≤ 0.05, ***P* < 0.01, ****P* < 0.001. **(E)** Flow cytometry analysis of KDM4c levels (HA AF-647, y-axis) and SOX2 levels (SOX2 AF-594, x-axis) in wild-type mESCs, and $KDM4c^{WT}$ cells (left). eGFP levels (y-axis) and KDM4c levels (x-axis) are also shown (right). **(F)** Epifluorescence images of embryonic bodies (EBs) without (+Dox) or with (-Dox) silencing at the reporter locus. Experimental outlines are shown below. **(G)** RT-qPCR analysis of RNA fold changes for Major Satellite Repeats, IAP elements, and L1mdTfI&II in WT, $KDM4c^{OE}$ overexpressing mESCs. *P*-values calculated by two-tailed unpaired Student's t-tests are indicated with asterisks. n.s., not significant (*P* > 0.05), **P* ≤ 0.05, ***P* < 0.01, ****P* < 0.001.

Figure S7

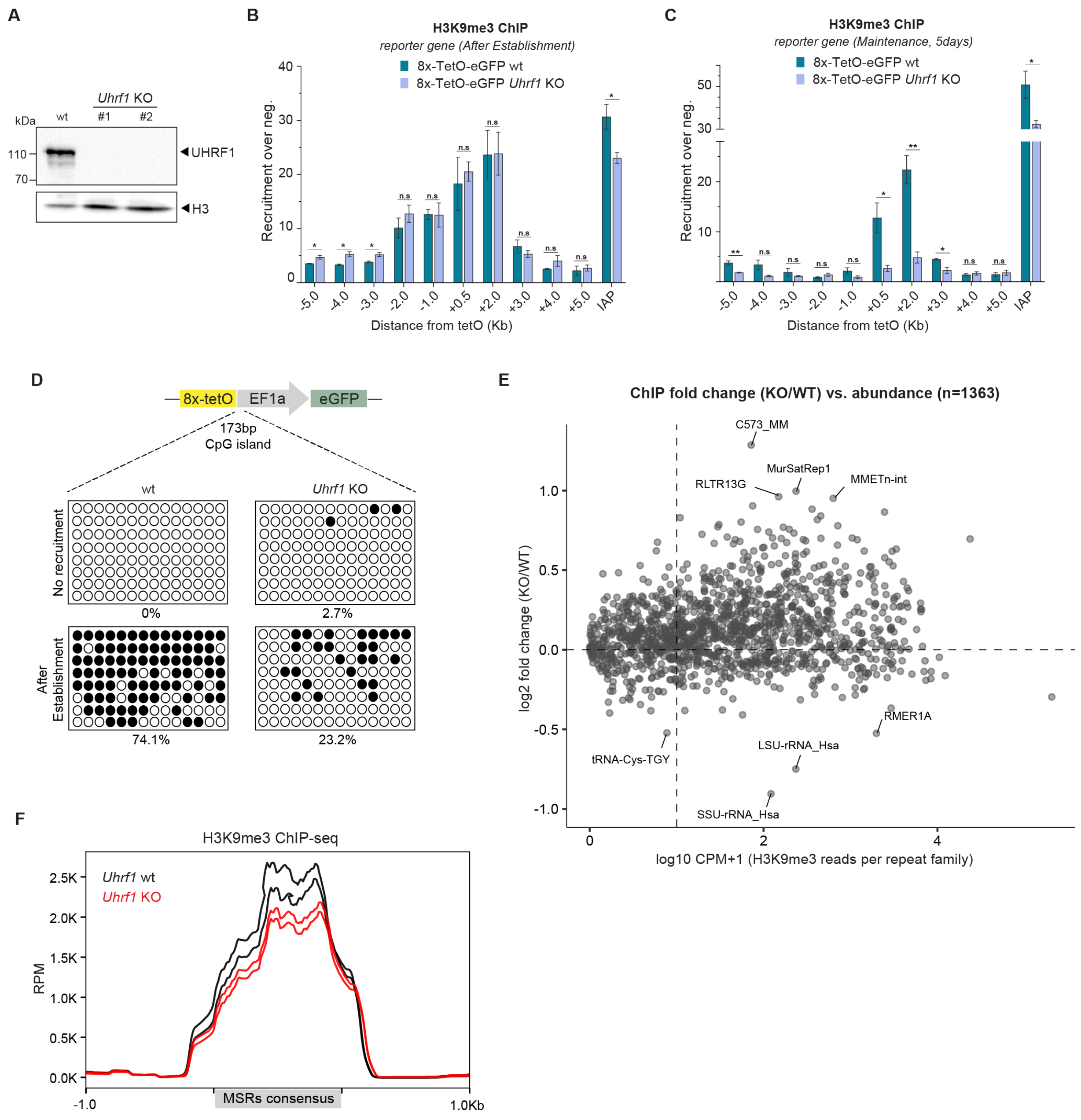

Figure S7. Deletion of *Uhrf1* diminishes epigenetic maintenance of H3K9me. Related to Figure 5.

(A) Western blot showing (top) protein levels of UHRF1 in *8x-tetO-eGFP* wild-type (wt) and two different clones (#1, #2) of *Uhrf1* KO mESCs and H3 western blot used as a loading control (bottom). Molecular weights in kilodalton are shown on the left. **(B)** ChIP-qPCR analysis for

H3K9me3 at the *8x-tetO-eGFP* reporter locus and surrounding regions in *8x-tetO-eGFP* wild-type (wt), and *Uhrf1* KO mESCs cultured in the absence (After Establishment, -Dox) of doxycycline. Values are shown relative to *Gapdh* used as a negative control region. Error bars, standard deviation (SD); n = 3 replicates. *P*-values calculated by two-tailed unpaired Student's t-tests are indicated with asterisks. n.s., not significant ($P > 0.05$), $^{*}P \leq 0.05$, $^{**}P < 0.01$, $^{***}P < 0.001$. **(C)** Same as in **B** but cells are cultured in the presence of doxycycline (+Dox) for 5 days during the maintenance phase. **(D)** Bisulfite sequencing of DNA CpG methylation at the reporter locus in *8x-tetO-eGFP* wild-type (wt), and *Uhrf1* KO mESCs cultured before establishment in the presence (No recruitment) or after establishment in the absence (After Establishment) of doxycycline. Filled and open circles represent methylated and unmethylated CpGs, respectively. Percentages (%) indicate methylated CpGs. **(E)** Scatterplot showing mean H3K9me3 read counts in wt mESCs (x-axis) for 1,363 repeat elements and $\log_2$ ratio of total H3K9me3 ChIP-seq reads (y-axis) per repeat element in *Uhrf1* KO (KO) versus wild-type (wt) mESCs. The top 4 increasing or decreasing repeats are highlighted. Source data for this figure are provided in Table S6. **(F)** Plot showing the normalized average density of H3K9me3 ChIP-seq across MSR consensus sequence in wild-type (wt) and Uhrf1 KO mESCs.

Figure S8

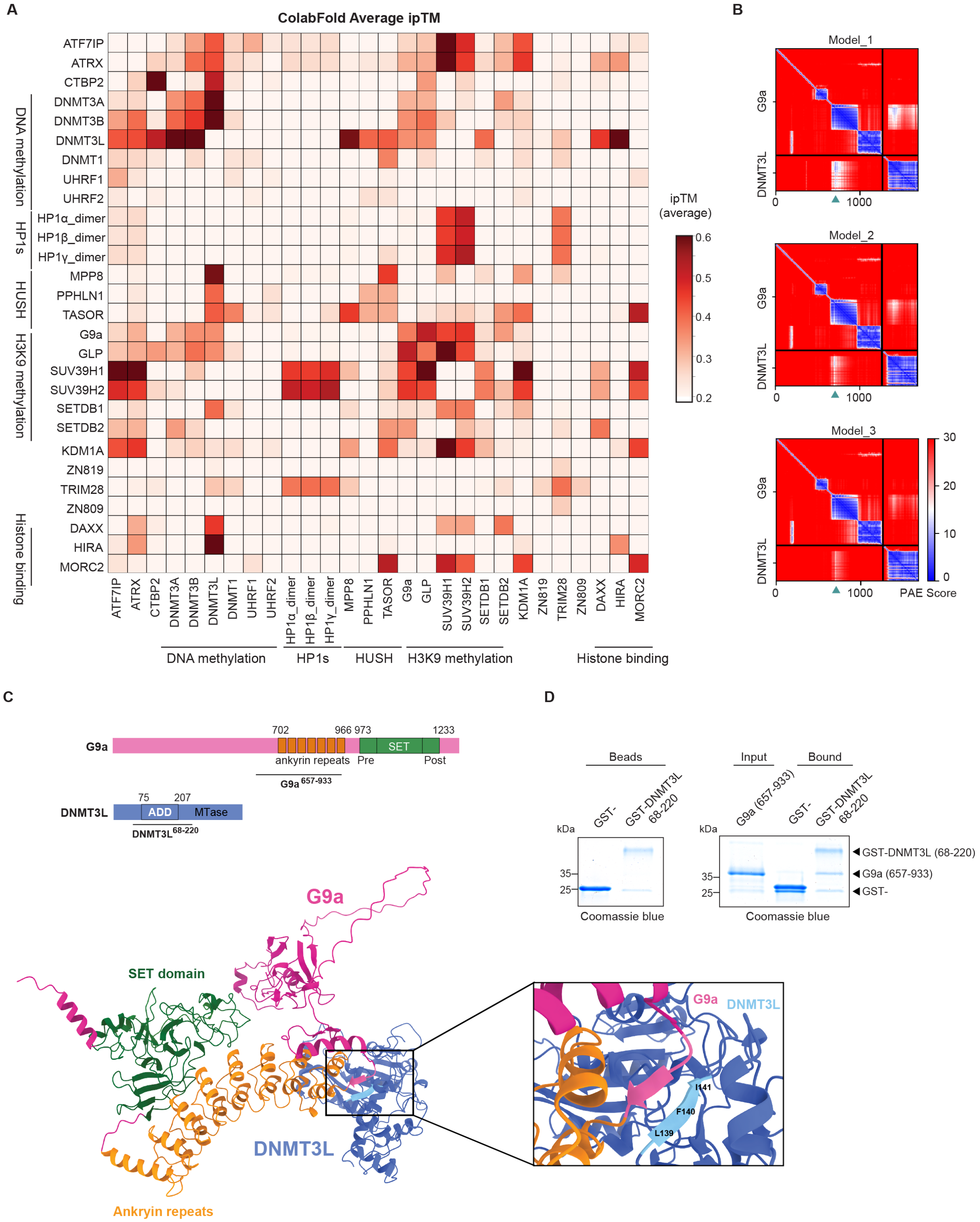
A
ColabFold Average ipTM
ATF7IP
ATRX
CTBP2
DNMT3A
DNMT3B
DNMT3L
DNMT1
UHRF1
UHRF2
HP1α_dimer
HP1β_dimer
HP1γ_dimer
MPP8
PPHLN1
TASOR
G9a
GLP
SUV39H1
SUV39H2
SETDB1
SETDB2
KDM1A
ZN819
TRIM28
ZN809
DAXX
HIRA
MORC2
DNA methylation
HP1s
HUSH
H3K9 methylation
Histone binding
ipTM (average)
0.6
0.5
0.4
0.3
0.2
B
Model_1
Model_2
Model_3
G9a
DNMT3L
0
1000
PAE Score
30
20
10
0
C
G9a
702
966 973
1233
SET
ankyrin repeats
Pre
Post
G9a 657-933
DNMT3L
75
207
ADD
MTase
DNMT3L 68-220
G9a
SET domain
DNMT3L
Ankryin repeats
G9a
DNMT3L
I141
F140
L139
D
Beads
Input
Bound
GST-
GST-DNMT3L 68-220
G9a (657-933)
GST-
GST-DNMT3L 68-220
kDa
35
25
kDa
35
25
GST-DNMT3L (68-220)
G9a (657-933)
GST-
Coomassie blue
Coomassie blue

Figure S8. AlphaFold Multimer screen for interactions between heterochromatin proteins. Related to Figure 6.

(A) Heatmap of average interface predicted template modeling (ipTM) scores of 28 mouse proteins (26 silencing factors and 2 ZNF proteins (ZNF809, ZNF819) expressed in mESCs) associated with the indicated functions. **(B)** Predicted alignment error (PAE) plots showing confidence of residue contacts for the full length DNMT3L with the full length G9a. PAE plots generated by 3 AF-M models for the complex of DNMT3L with G9a. The peptide regions around the ankyrin repeats domain of G9a that are predicted to interact with DNMT3L are indicated with teal arrowheads. **(C)** Schematic representation of domain organization of *M. Musculus* G9a (top) and DNMT3L (bottom) proteins. Bold lines indicate G9a protein ($G9a^{657-933}$) and DNMT3L protein ($DNMT3L^{68-220}$) used for GST-based binding assays. **(D)** Predicted structure depicting interaction peptides between G9a and DNMT3L. The interaction between two antiparallel β-sheets of the two proteins is highlighted (G9a, magenta; DNMT3L, light blue). **(E)** Binding assays testing the interaction between $G9a^{657-933}$ with GST- or GST-$DNMT3L^{68-220}$. Arrowheads indicate the proteins used in the assay.

Figure S9

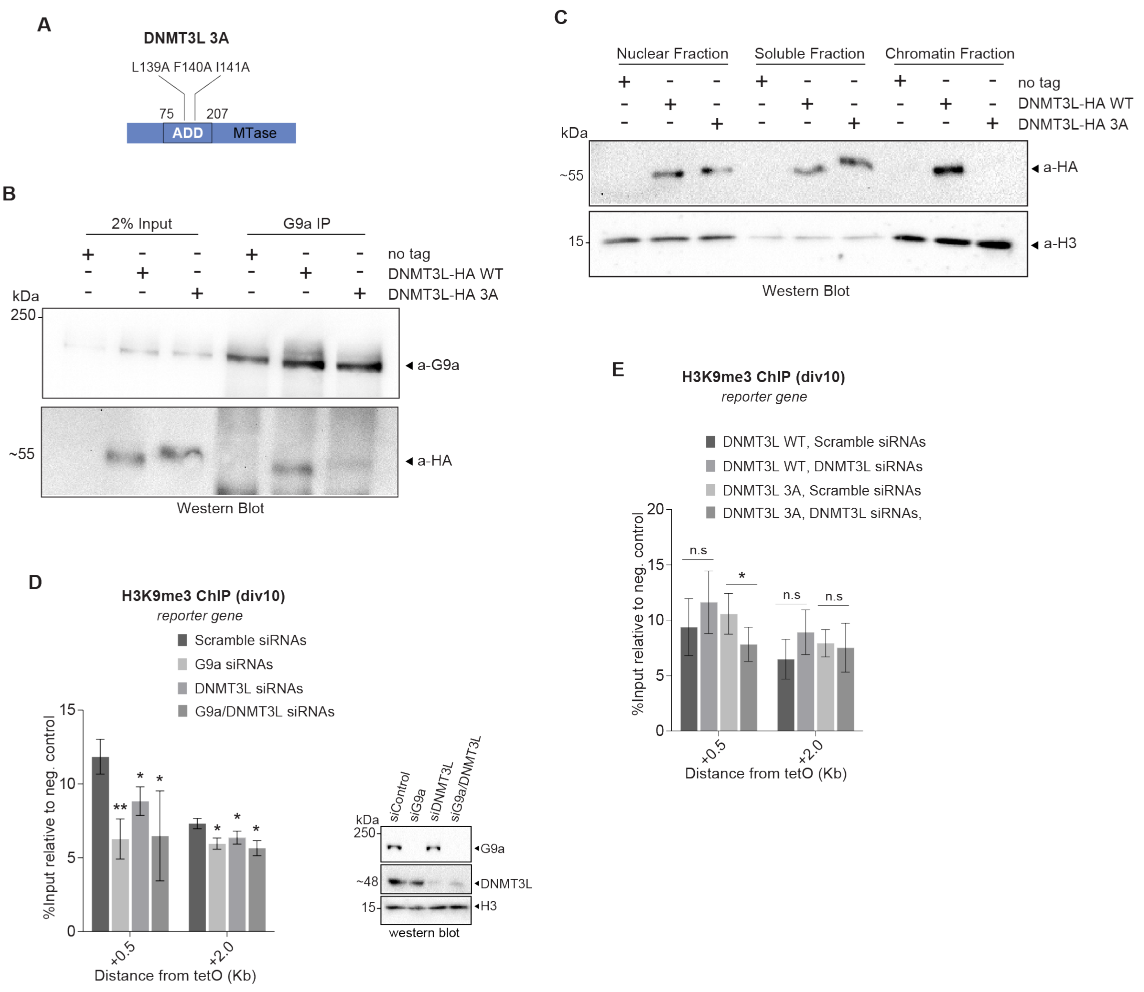

Figure S9. *In vivo* interaction of G9a and DNMT3L proteins. Related to Figure 6.

(A) Diagram highlighting domain organization of DNMT3L. DNMT3L 3A denotes DNTM3L with L139A, F140A, I141A substitutions. **(B)** *In vivo* G9a immunoprecipitation in mESCs not expressing tagged DNMT3L, expressing HA-tagged DNMT3L WT, or DNMT3L 3A. **(C)** Western blot showing the levels of DNMT3L WT and DNMT3L 3A in total nuclear, soluble and chromatin fractions. **(D)** ChIP-qPCR analysis for H3K9me3 at the *8x-tetO-eGFP* reporter locus in mESCs transfected with control or siRNAs targeting G9a, DNMT3L, or both, following the outline in panel **Figure S4G**. Values are normalized to *Gapdh* promoter region. Error bars, standard deviation (SD); n = 3 replicates. *P*-values calculated by two-tailed unpaired Student's t-tests are indicated with asterisks. n.s., not significant ($P > 0.05$), *$P \leq 0.05$, **$P < 0.01$, ***$P <$ 0.001. **(E)** ChIP-qPCR analysis of H3K9me3 at the *8x-tetO-eGFP* locus in mESCs expressing

siRNA-resistant DNMT3L WT or DNMT3L 3A transfected with control or siRNAs targeting endogenous DNMT3L. Values are normalized to *Gapdh* promoter region. Error bars, standard deviation (SD); n = 6 replicates. *P*-values calculated by two-tailed unpaired Student's t-tests are indicated with asterisks. n.s., not significant ($P > 0.05$), *$P \leq 0.05$, **$P < 0.01$, ***$P < 0.001$.
